# Supplementary material for: Semiconductor room-temperature maser
Source: Nat Commun. 2026 Jul 25;17:7267. doi: 10.1038/s41467-026-75446-2 (PMC13400640; doi:10.1038/s41467-026-75446-2)
Supplement: Supplementary file 1 — Supporting Information [file 41467_2026_75446_MOESM1_ESM.pdf]

Supplementary Information for:

Semiconductor Room-Temperature Maser

Andreas Gottscholl[1,2,a)], Maximilian Wagenhöfer[1], Valentin Baianov[1], Emilian Eisermann[1], Vladimir Dyakonov[1], Andreas Sperlich[1,b)]

[1]Experimental Physics 6 and Würzburg-Dresden Cluster of Excellence ctd.qmat, Julius-Maximilians-Universität Würzburg, 97074 Würzburg, Germany

[2]NASA Jet Propulsion Laboratory, California Institute of Technology, Pasadena, California, USA

[a)] Electronic mail: gottscholl.andreas@gmail.com

[b)] Electronic mail: andreas.sperlich@uni-wuerzburg.de

Setup Details

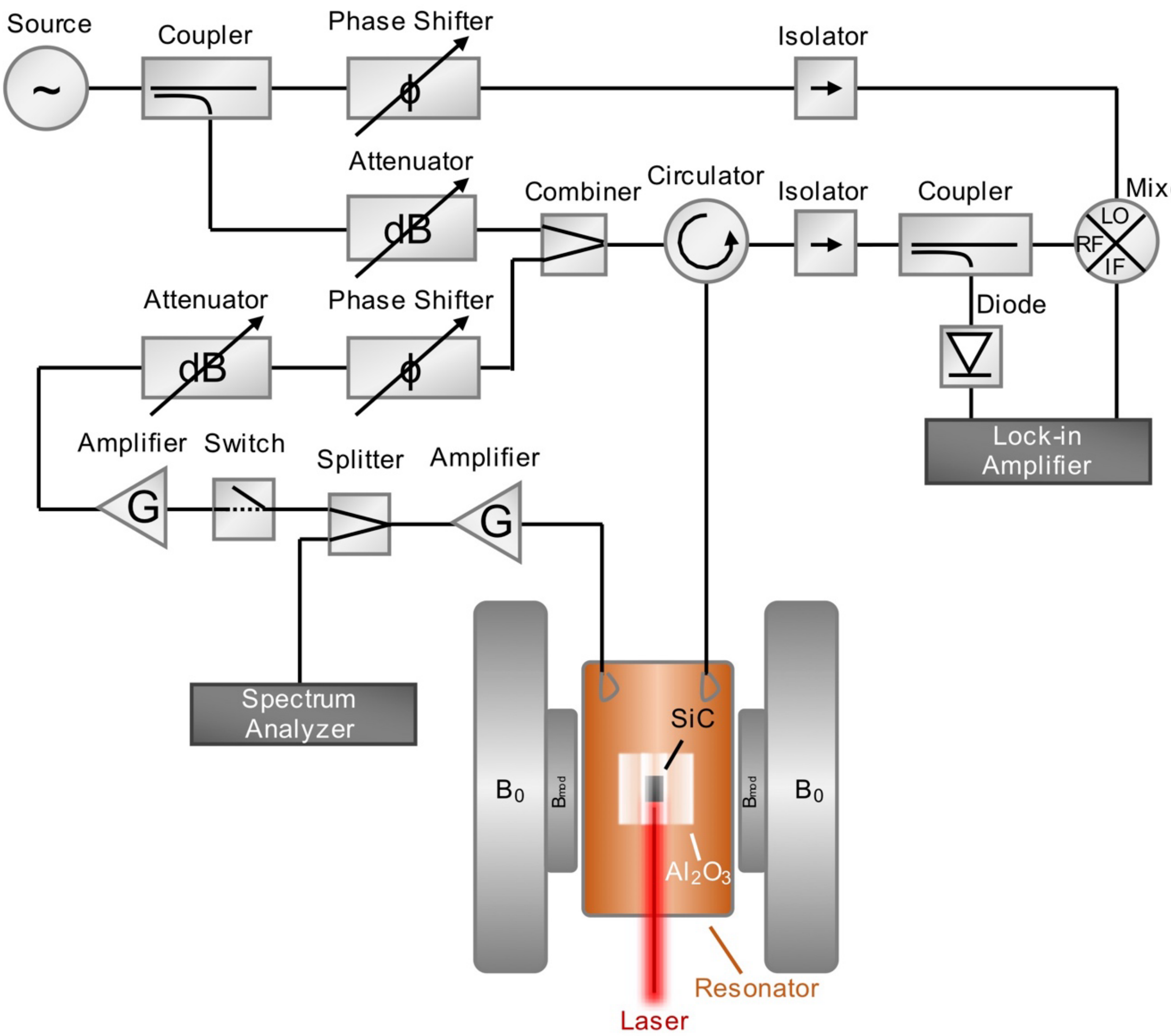

Supplementary Figure 1. Self-built setup for Q-factor, electron paramagnetic resonance, (Q-boosted) maser, amplifier and refrigerator measurements.

The main results of this work were all recorded with one single self-built setup. Since the here-reported microwave bridge enables six different modes of operation, we will focus on the different signal paths separately for each measurement in the next sections. The whole setup is illustrated in Supplementary Fig. 1. As a microwave source we used an *Anritsu MG 3694C*. Most of the components are commercially available: As coupler we used a -10 dB *Mini-Circuits ZUDC10-02183-S*+ (left) and a -20 dB *Radiall R433724700* (right). The isolators *Aerotek I2E1L1FF* are integrated to block unwanted microwave reflections. For combiner/splitter we used *Mini-Circuits ZFRSC-183-S+,* while the detection is performed with a detector diode *Advanced Control Components ACTP-1504P*, a mixer *Marki M10412LA*, a lock-in amplifier *Signal Recovery 7230* and a spectrum analyzer *WSA 5000* (see the following section for detailed measurement explanations). To compensate microwave losses, we used a *Miteq AFS4.08001200-10.200* LNA (right) and a *Narda N62448-243* (left). A microwave switch *Mini-Circuits MSP2TA-18-12*+ as well as two modified Rotary Vane attenuators with added servo motors are controlled via an *Arduino*. For phase corrections we used two analog phase shifters from an old *Bruker ER047* microwave bridge, which are controlled via the DAC of the lock-in amplifier. For optical pumping we used an 808 nm-laser *K808FANFA-15.00W* from *BWT* which provides a variable power of 0 – 15000 mW. The resonator is placed in a cryostat with an optical window on the side. For low temperatures, the cryostat walls are filled with liquid nitrogen, and the cryostat volume is maintained in a helium atmosphere to efficiently transfer the temperature to the resonator and the sample. The temperature is monitored using a *Cernox* temperature sensor attached to the exterior of the resonator. The microwaves are coupled in and out of the resonator via loop antennas.

Q-factor measurement

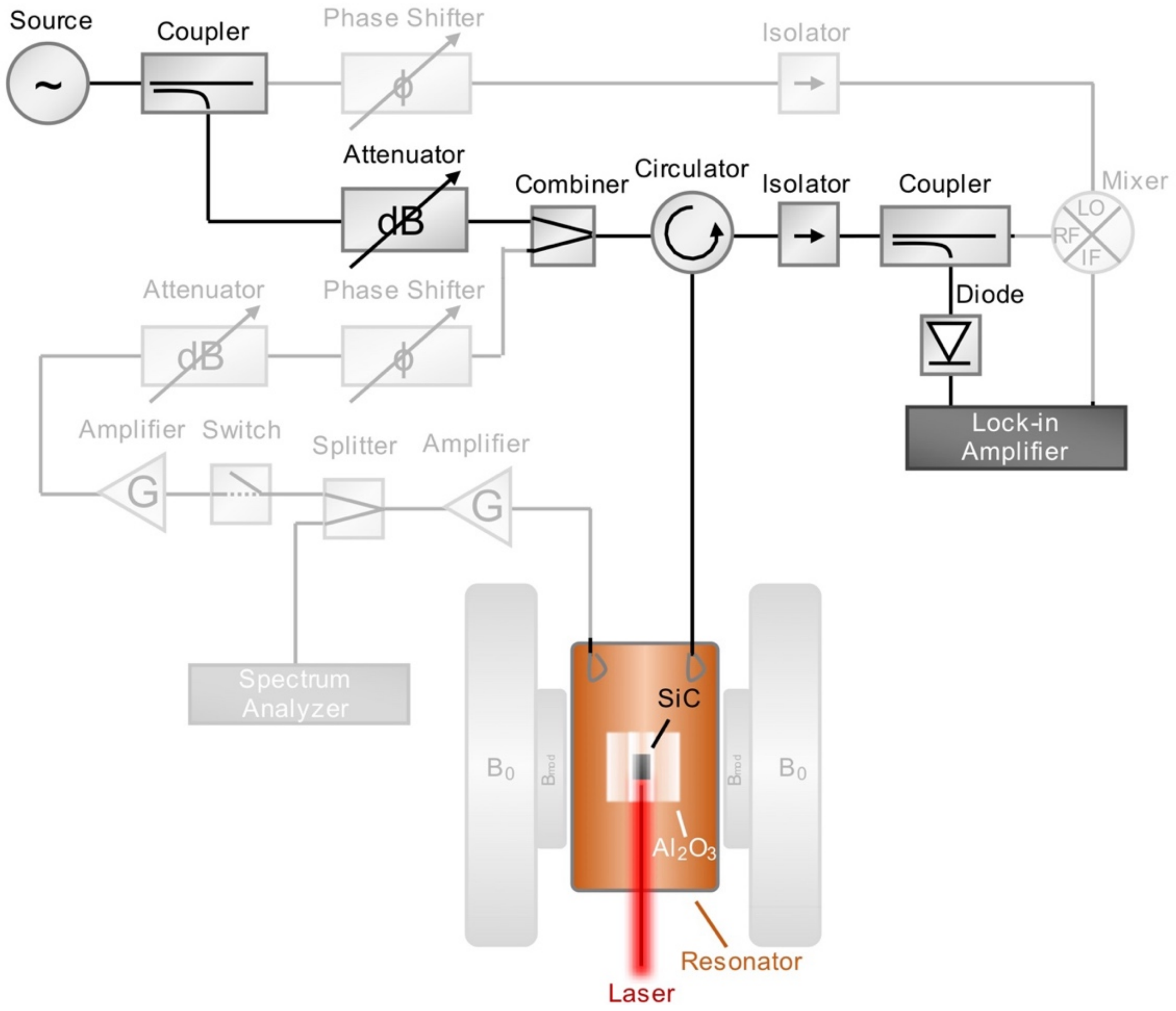

Supplementary Figure 2. Setup for Q-factor measurements. The microwave frequency is swept while the output is on/off-modulated. The reflected microwave power is measured via a detector diode and a lock-in amplifier.

The basis for all measurements reported in this paper is the determination of the Q-factor of the resonator and in particular its resonance frequency. This is accomplished by sweeping the frequency of an inserted microwave probe while measuring the reflected microwave power. Via a circulator the reflected microwaves are separated from the inserted microwaves and detected with a detector diode. Since the measurement provides a very high signal-to-noise ratio only a very small amount of the signal is required. Thus, we use a coupler with -20 dB which transmits most of the signal for the EPR measurement (see next section). The detection of the small signal is realized with a lock-in amplifier. We choose an on/off modulation of the microwaves in order to observe the reflection directly, which results in a quantitative analysis of the Lorentzian dip providing information about under/over-coupling or critical coupling of the resonator (see Supplementary Fig. 9). This calibration routine for the maser/amplifier/refrigerator is performed with an active laser output since a small heating input leads to a drift of the resonance frequency. Thus, we start with further measurements as soon as the Q-factor measurements reveals a stable resonance frequency.

Electron Paramagnetic Resonance

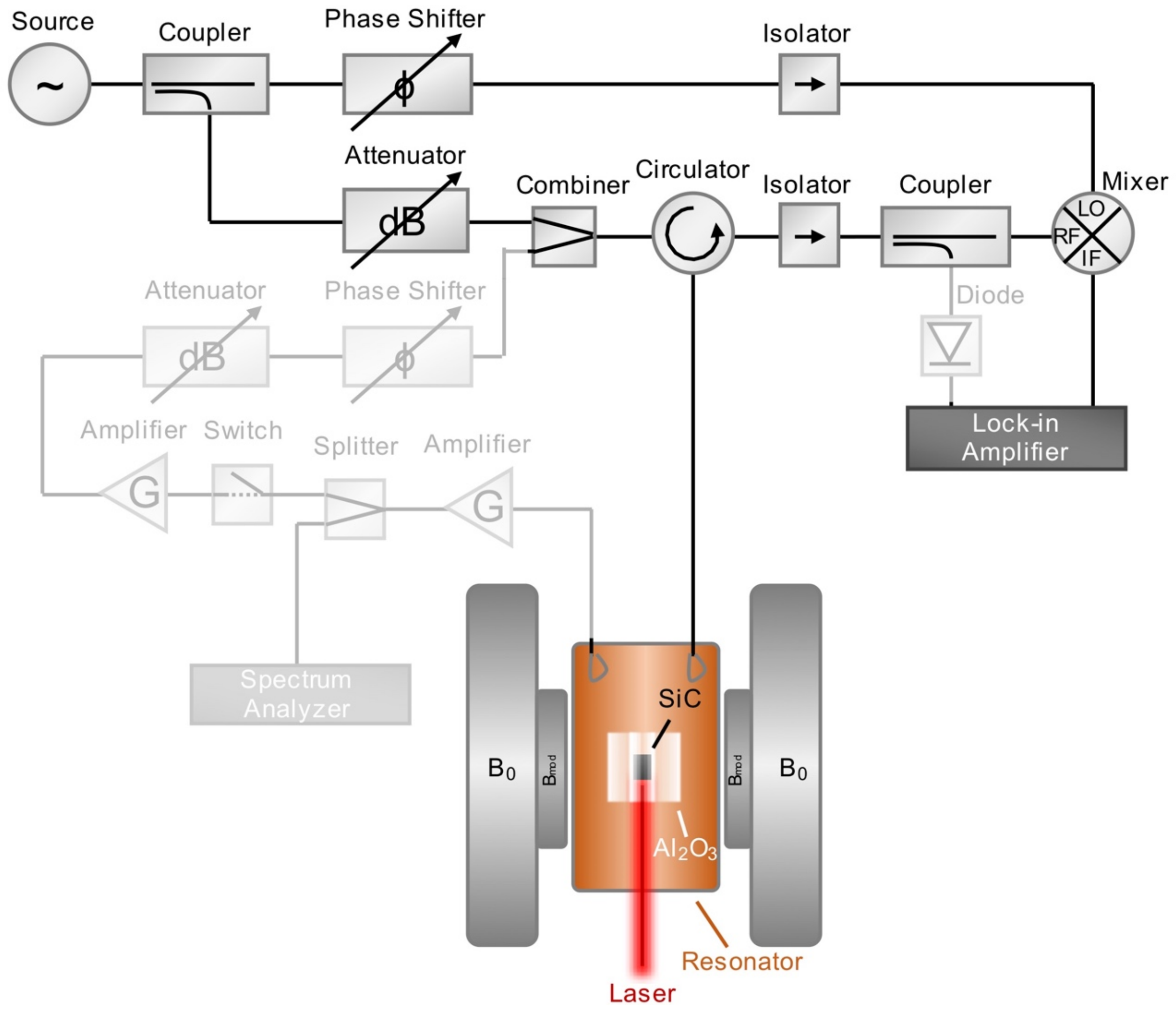

Supplementary Figure 3. Setup for electron paramagnetic resonance. The microwave frequency is fixed to the resonance frequency of the resonator. The main part of the microwaves drives phase-adjusted the local oscillator of a mixer. A small part is attenuated to a sufficiently low power and interacts with the sample. The remaining microwaves are detected via the mixer and the lock-in amplifier.

With the previous measurement we extracted the correct frequency regime ($f \approx f_R$). Besides the frequency, a maser/amplifier/refrigerator requires also the correct magnetic field range ($B \approx B_\pm$). A perfect measurement for probing the resonance condition of the spin system is electron paramagnetic resonance (EPR). Again, the microwave reflection is measured, but the swept quantity is the magnetic field using a pair of Helmholtz coils (see Supplementary Fig. 3). The microwave output is fixed to the resonance frequency f_R in a continuous wave mode. To enhance the signal-to-noise ratio we are using the same lock-in amplifier (second input), and the magnetic field is sinusoidally modulated with a pair of small modulation coils. The interacting microwaves are attenuated (0 – 60 dB) to avoid saturation of the spin system.

The main part of the microwaves is guided through the first coupler to drive the local oscillator of the mixer. A phase shifter is implemented to phase-adjust the microwaves, thus providing the correct phase of the down-converted intermediate frequency. The remaining

down-converted frequency is identical to the modulation frequency and recorded by the lock-in amplifier. Besides the information of the resonant magnetic field ($B \approx B_{\pm}$), EPR reveals the spin polarization of the system (see [1,2] for details). Thus, the pump threshold for the maser can be estimated with EPR.

Maser

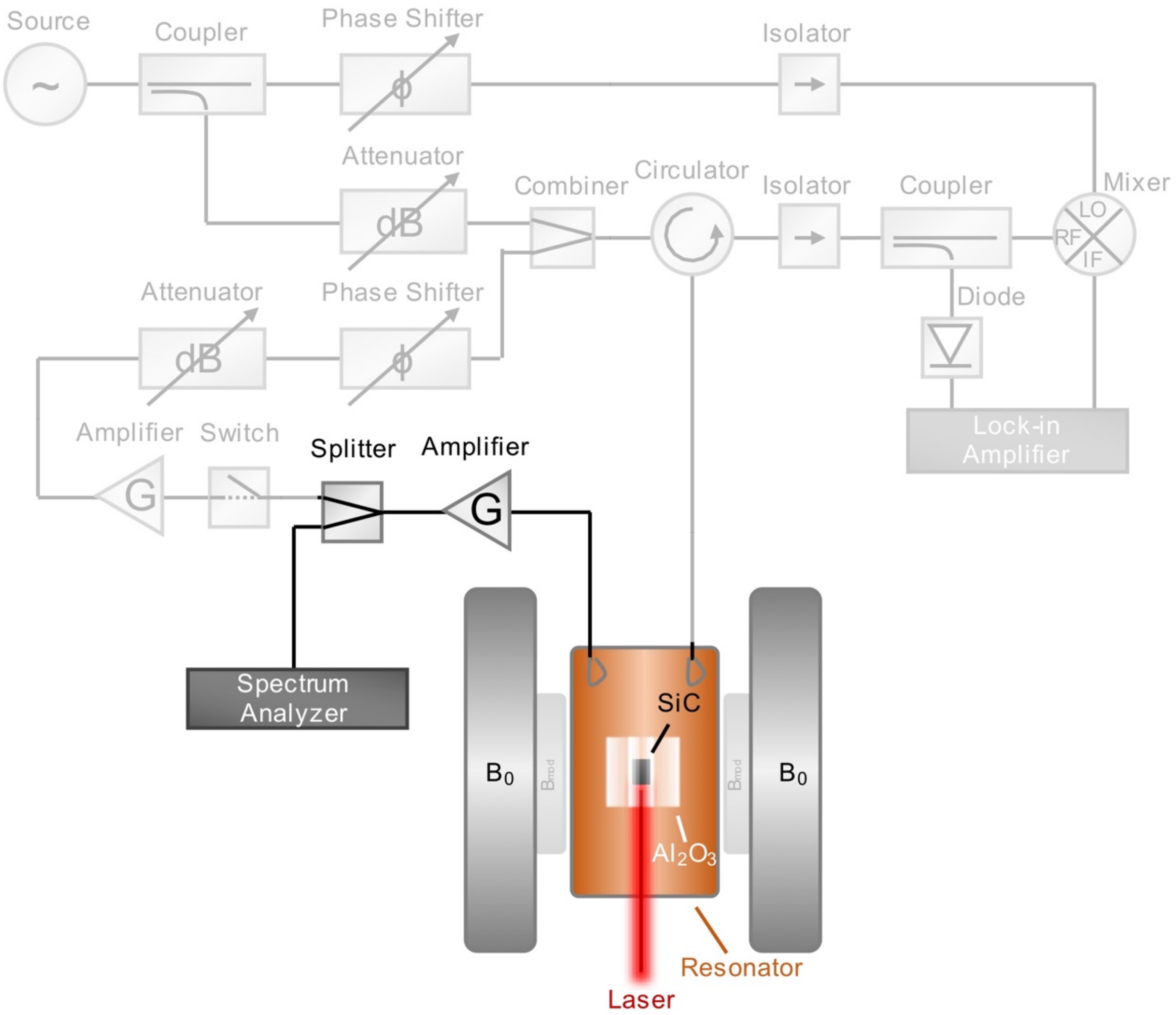

Supplementary Figure 4. Setup for maser measurements. The sample is excited via a laser within an external magnetic field. The emitted microwaves are amplified (to compensate losses) and guided to a spectrum analyzer.

For the maser output measurement, the magnetic field range is arranged according to the previous EPR experiment, and the frequency range of the recording spectrum analyzer is adjusted to the resonance frequency of the resonator. To compensate several losses (e.g. inserted splitter for the next measurement type), a LNA is inserted.

Q-boosted Maser

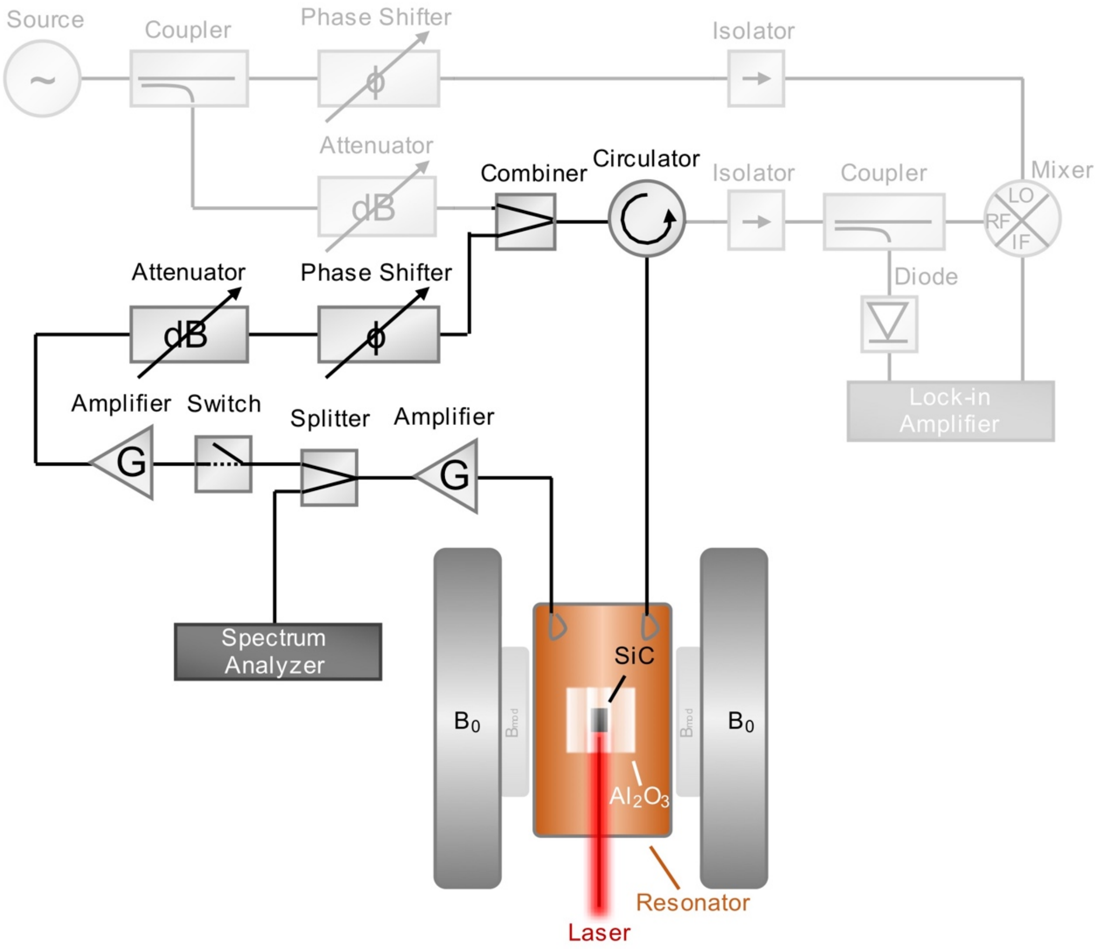

Supplementary Figure 5. Setup for Q-boosted maser measurements. By closing the microwave switch, the feed-back loop can be activated. After amplification (+attenuation) and phase adjustment the microwaves are inserted back into the resonator. Only a part of the signal is guided to the spectrum analyzer.

The artificial enhancement of the Q-factor is realized by a feed-back loop. The Q-boosting is activated by closing the microwave switch. 50% of the output signal after the LNA are amplified by a second amplifier. To avoid an overdrive the gain is controlled by attenuating (0 – 60 dB) the previously amplified signal. After phase adjustment via a phase shifter, the microwaves are inserted back into the resonator.

Amplifier/Refrigerator

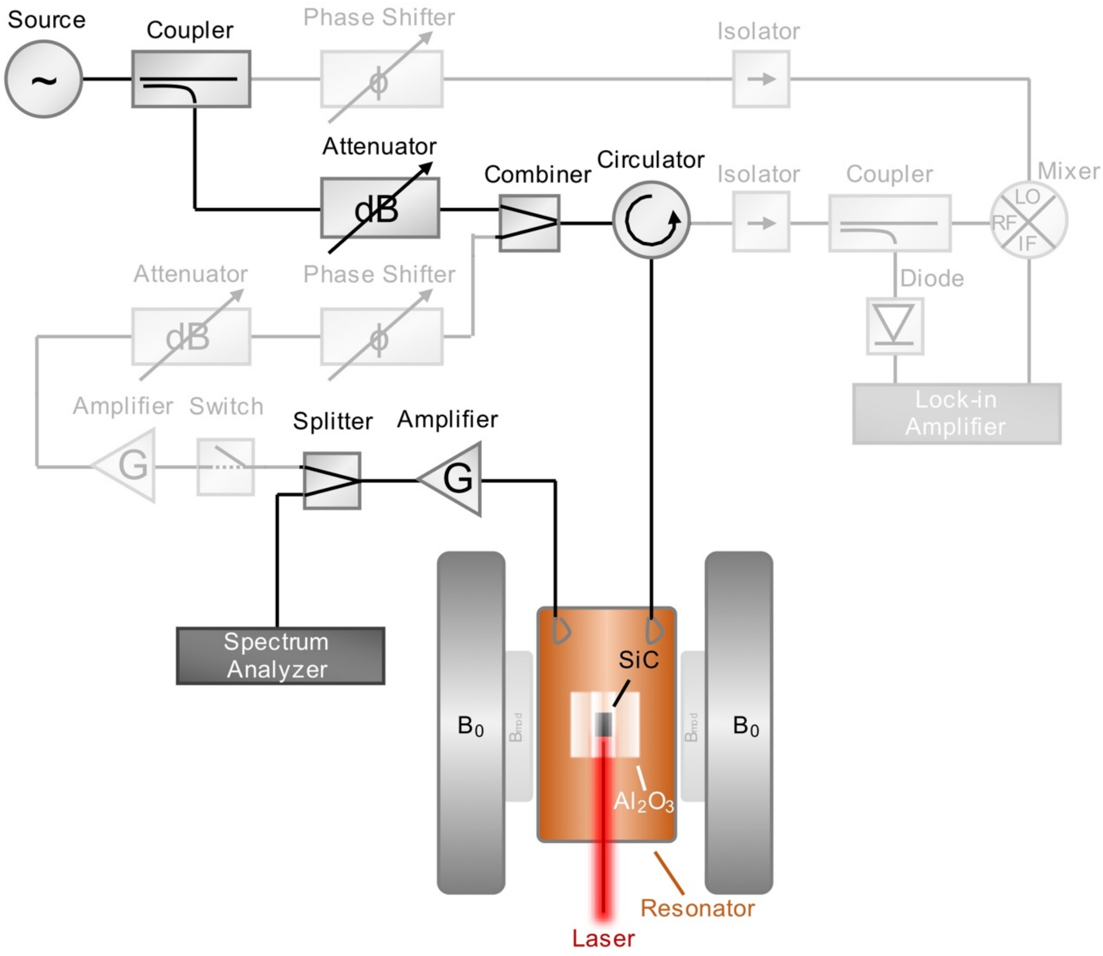

Supplementary Figure 6. Setup for amplifier/refrigerator measurements. An arbitrary microwave signal is inserted into the resonator by one antenna. A second antenna detects the output signal. The amplifier (refrigerator) can be switched on for $B = B_+$ ($B = B_-$) and off for $B \neq B_+$ ($B \neq B_-$), respectively.

The amplifier and refrigerator are analyzed by inserting a test signal into the system. The frequency is adjusted to the resonance frequency and the magnetic field within the range of the resonant condition. The amplifier (refrigerator) can be switched off by an off-resonance magnetic field $B \neq B_+$ ($B \neq B_-$). As soon as the magnetic field is in the range of the resonance condition $B = B_+$ ($B = B_-$), the spin system interacts with the microwaves, and the amplification (absorption) is recorded with the spectrum analyzer.

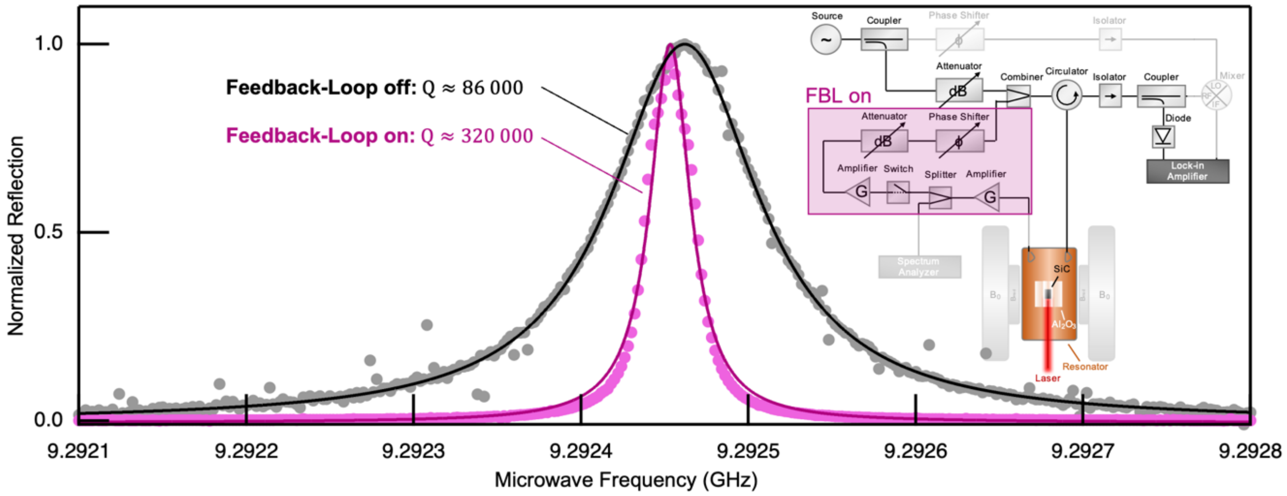

Supplementary Figure 7. Effect of the activated feedback loop on the Q-factor. A portion of the extracted microwave signal is amplified and fed back into the system to interfere constructively with the circulating microwaves. A phase shifter within the loop ensures constructive interference, while a variable attenuator positioned after the amplifier controls the overall gain of the feedback loop.

Resonator Design

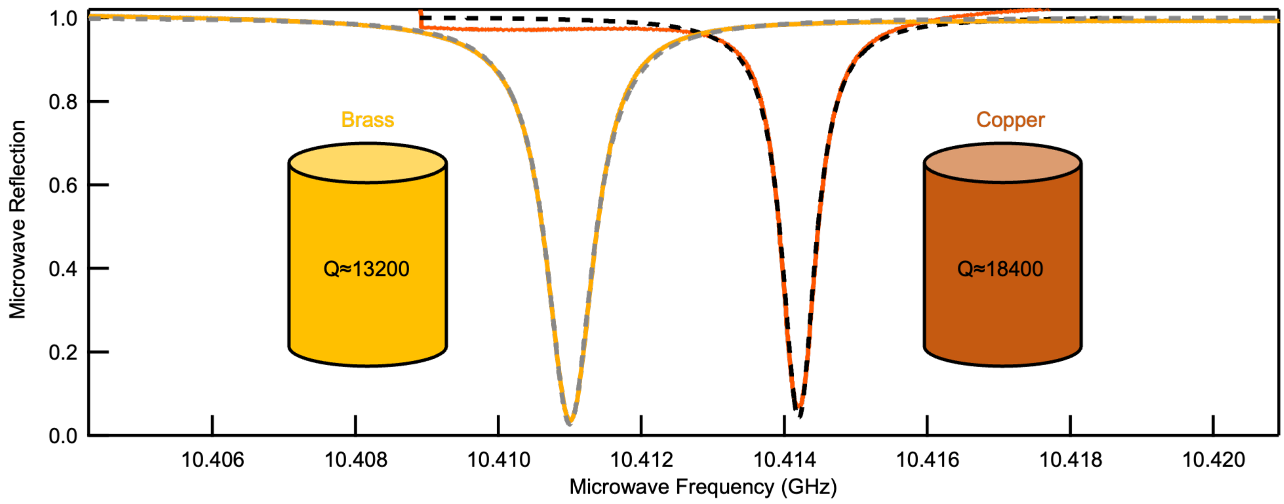

Supplementary Figure 8. Influence of the resonator material on the resonator Q-factor. Due to the higher conductivity, the ohmic losses are reduced in a copper resonator in comparison to a brass resonator with identical dimensions. The Q-factor values are shown here for an empty cavity (with sapphire, but without silicon carbide).

Different parameters were varied iteratively to find the most suitable resonator for the maser/amplifier/refrigerator. We started with an empty brass and copper resonator with dielectric sapphire core and compared the influence of conductance on the Q-factor (see Supplementary Fig. 8). As expected, the copper resonator provides a higher value. Therefore, we continued with height-dependent measurements (see Supplementary Fig. 9 a and b). Here, we already included the gain material to find the best constellation for a SiC-based device. We further changed the diameter of the resonator with the highest Q-factor

from the height-dependent measurements to get the highest value of Q = 17600 (see Supplementary Fig. 9). To increase the Q-factor even more we can either under couple the system or cool down the resonator to reduce ohmic losses (see Supplementary Fig. 10). The highest value of Q_{max} = 85000 was reached for a temperature of T = 87 K. This was approximately the lowest possible temperature of the liquid nitrogen cryostat, since the whole ensemble has to be cooled resulting in thermal losses. However, as soon as the laser is activated the temperature stabilizes around T = 110 K.

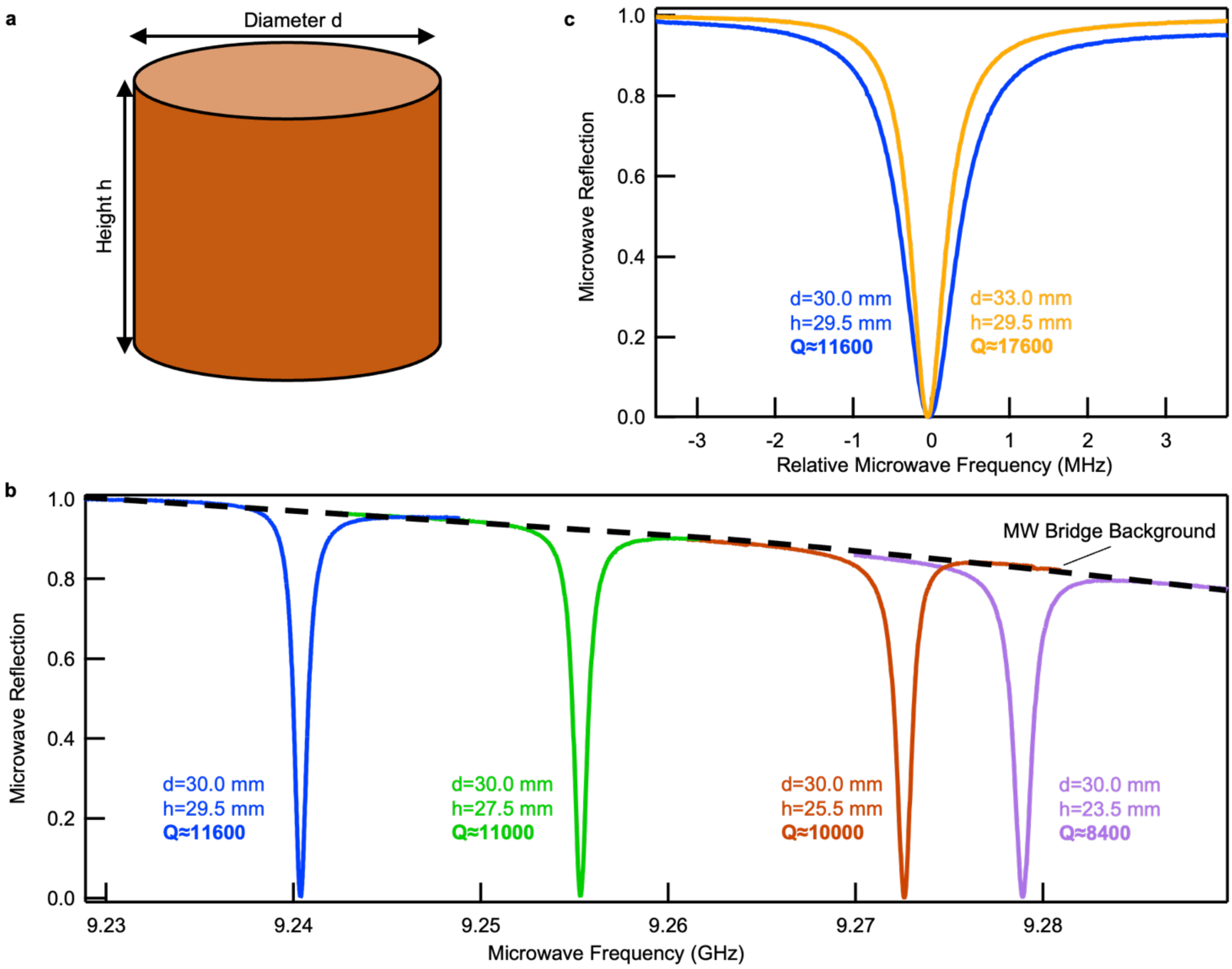

Supplementary Figure 9. Influence of the resonator dimensions on the Q-factor. a The height h is varied as well as the diameter d of the copper resonator filled with silicon carbide in order to find the highest Q-factor. **b** First, the height is varied. With increasing height, the resonance frequency shifts towards lower frequencies (absorption due to the microwave bridge background is indicated with a dashed line). **c** We chose the resonator with the highest Q-factor and enhanced the value by changing the diameter (relative frequency used due to large resonance frequency shift of 78 MHz).

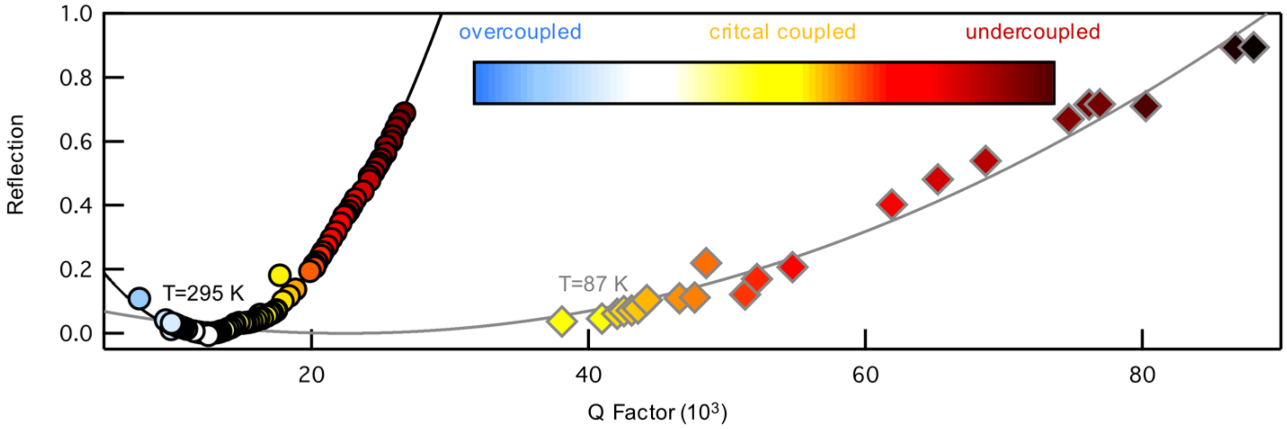

Supplementary Figure 10. Quality factor variation of the resonator without feedback loop. The Q-factor can be varied slightly by moving the coupling antenna in and out of the resonator. Depending on an over coupled (blue), critically coupled (yellow) and under coupled (red) state, the resonator reaches values up to 30000 at room temperature (black circles) and 85000 at cryogenic temperatures (grey diamonds). To study resonators with even higher values an artificial enhancement (feedback-loop) is required.

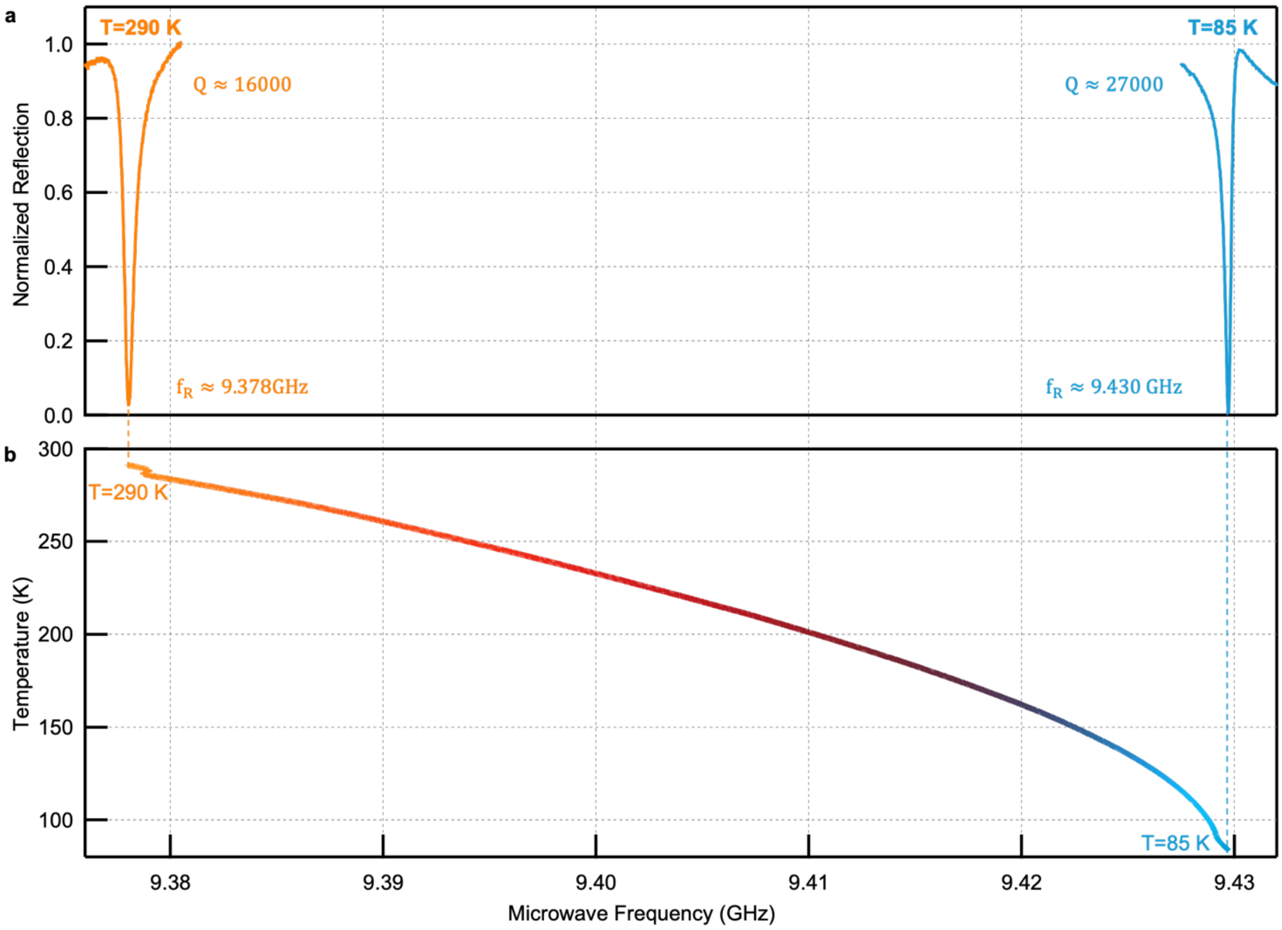

Supplementary Figure 11. Temperature dependence of the resonator. The resonance frequency is shifting by approximately 50 MHz within a temperature range from 85 – 290 K due to thermal expansion. The Q-factor is measured at 290 K and 85 K. Between the two resonances, an automatic frequency control locks the resonance frequency of the resonator and provides intermediate frequencies for the whole temperature range.

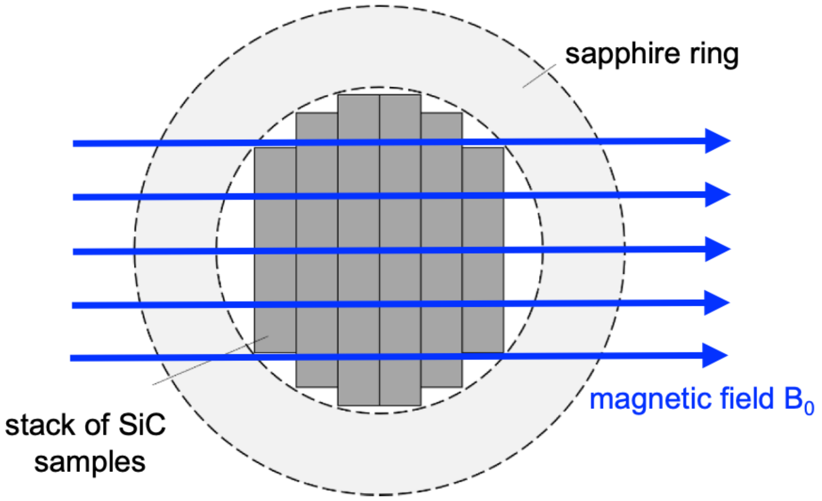

Supplementary Figure 12. Sample geometry within the resonator. In order to achieve a high filling factor we use a stack of rectangular SiC samples. Notably, the size varies to fit into the cylindrical sapphire ring. We chose this rectangular design instead of having round SiC discs to ensure a parallel magnetic field with respect to the crystal axis. The magnetic field would be perpendicular for SiC discs, leading to a 50% population inversion reduction.[3]

Maser/Amplifier simulation and experimental data

Simulations according to [4] were performed with the following parameter set:

Parameter	Value	Source	Diamond Maser Values [7]
γ_{eg}	26 s^{-1}	$T_1^{-1}(T = 110$ K)	208 s^{-1}
N	$2.19 \cdot 10^{13}$	see Figure S11	$4 \cdot 10^{13}$
ω_c	$2\pi \cdot 9.3$ GHz	Q-factor measurement	$2\pi \cdot 9.2$ GHz
T_2^*	250 ns	based on EPR	500 ns
g	$2\pi \cdot 0.05$ Hz	see Figure S12	$2\pi \cdot 0.11$ Hz
P	1 – 10000 mW	laser power range	400 mW
Q	10^4-10^6	FB-loop range	$3 \cdot 10^4$

γ_{eg}: The value of 26 s^{-1} is extracted from [5] for a temperature of $T = 110$ K.

N: The number of participating spins is determined by dark EPR. The corresponding measurement is depicted in Figure S13. A piece of the SiC wafer (irradiated with 2MeV electrons with a fluence of $2{\cdot}10^{17}$ cm^{-2}) was investigated in a Magnettech ESR5000 at room temperature (Figure S13 a). We used the left transition (Figure S13 b) and compared the double integral (area of the bottom plot) with the value of a reference sample with a known spin number (BDPA in Supplementary Fig. 13c: $n = 3.6{\cdot}10^{17}$). Thus, we extracted a spin number of $2.7{\cdot}10^{12}$ for the SiC sample resulting in a spin density of $2.27{\cdot}10^{15}$ cm^{-3}. The total volume of the sample used for the maser/amplifier/refrigerator is 0.0344 cm^3 resulting in a total spin number of $7.8{\cdot}10^{13}$. The spin number for one transitions (see Figure 1b) is reduced to $\frac{1}{2} \cdot 56.1\% \cdot 7.8 \cdot 10^{13} = 2.19 \cdot 10^{13}$ taking the two transitions (B_+ and B_-) and the isotopic ratio of silicon (see [3] for detailed discussion) into account.

ω_c: The value of $2\pi \cdot 9.3$ GHz is based on Q-factor measurements. Small variations can be observed between the measurements, however, the difference is in the MHz regime.

T_2^*: To access the coherence time in the setup used here, we analyzed the linewidth of the system with continuous-wave (CW) electron paramagnetic resonance (EPR). Notably, pulsed EPR measurements are not applicable here, as the Q-factor is too high for such experiments, leading to a long ring-down time of several microseconds. Therefore, we determined the T_2^* time using the natural linewidth. We observed a linewidth of approximately 45 µT, which can be attributed to T_2^* as there is no evidence of saturation due to overmodulation or power broadening. This corresponds to a frequency linewidth of 1.3 MHz, which in turn indicates a coherence time of approximately 250 ns. This value agrees with those reported by [6], where a T_2^* time of 200 – 300 ns was observed in natural SiC at a similar irradiation dose.

g: Based on the resonator design, the coupling strength is expected to lie within the range $g/(2\pi) =$ 0.02 Hz – 0.11 Hz. [4] This value is influenced by numerous parameters and is not straightforward to determine directly. Therefore, we treated it as the sole free parameter in our model and varied it accordingly. As a reference point for the maser threshold, we used the maser measurement with the lowest output power (Figure 1c), since all other measurements yielded significantly higher maser outputs. By varying the coupling g, we observed corresponding shifts in the maser threshold along the Q-factor axis. Representative examples are shown in Supplementary Fig. 15. The best agreement with the experimental data was obtained for $g = 2\pi \cdot 0.05$ Hz, which is consistent with values reported by [4].

P: The value of the laser power is swept in the range of 1 mW and 10 W since this is within the experimental achievable range (*K808FANFA-15.00W* laser from *BWT*).

Q: The Q-factor axis was plotted for 10^4 – 10^6. Values of 10^4 – 10^5 are reached by an under coupling of the resonator (see Supplementary Fig. 9). Values exceeding 10^5 are only possible via a feed-back loop which can be automatically activated and controlled by a microcontroller.

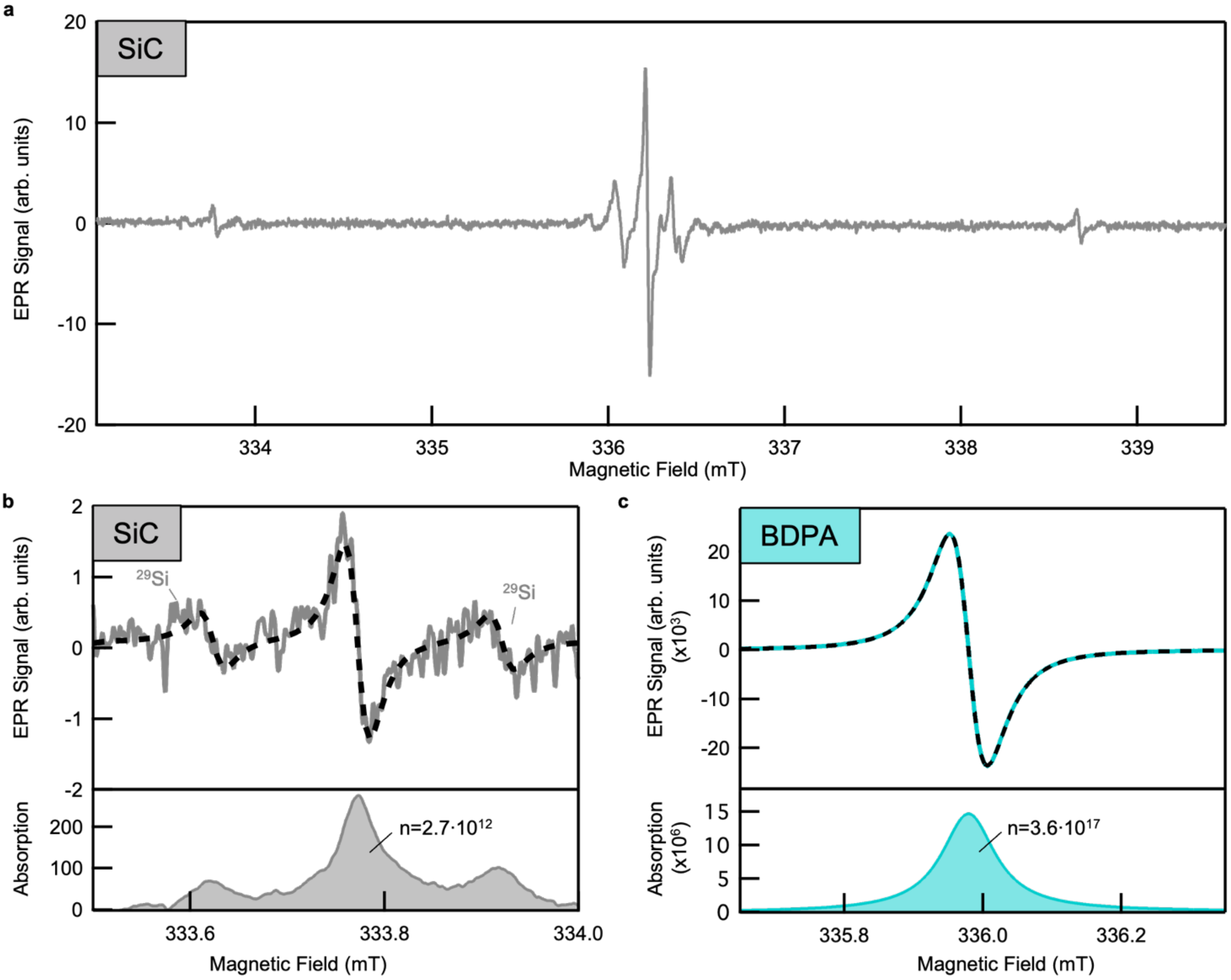

Supplementary Figure 13. Quantitative EPR measurements determine the absolute spin number. **a** Room temperature continuous wave EPR measurement without illumination of a SiC sample used in this paper. **b** Zoom into the left transition in order to get only the contribution of the V2 defect. The double integral is proportional to the absolute spin number n. **c** EPR spectrum of a reference sample (BDPA) with known spin number. All measurements were performed in a Magnettech ESR5000.

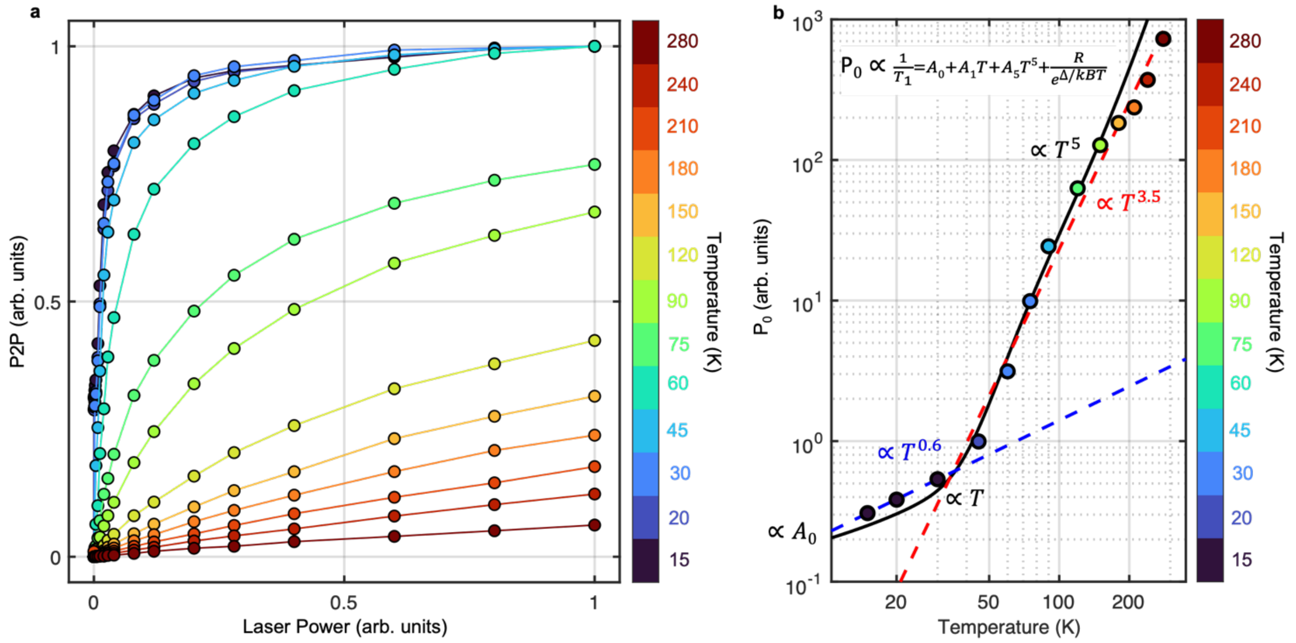

Supplementary Figure 14. Temperature-dependent characteristic pump power measured via EPR (see [1] for details). Measurements were performed using a home-modified Bruker E300. **a** Peak-to-peak amplitude of the EPR signal as a function of temperature and laser power. Fitting this behavior using parameters from [1] yields the characteristic pump power. **b** Extracted characteristic pump power as a function of temperature. The trend closely follows the known spin-lattice relaxation rate (shown in black) $\frac{1}{T_1} = A_0 + A_1 T + A_5 T^5 + \frac{R}{e^{\Delta/k_B T}}$ (see [5] for details). A quantitative analysis reveals minor deviations in the power-law dependence. Fits for the low- and high-temperature regimes are shown in blue and red, respectively, with their intersection marking the fit regime. At low temperatures, the regime approaches the A_0 plateau, which limits the fitting accuracy. At high temperatures, the dominant $\frac{1}{T_1} \propto T^5$ dependence is reduced due to the influence of continuous optical pumping on the relaxation time. Consequently, the temperature dependence is less pronounced.

Further measurements and simulations

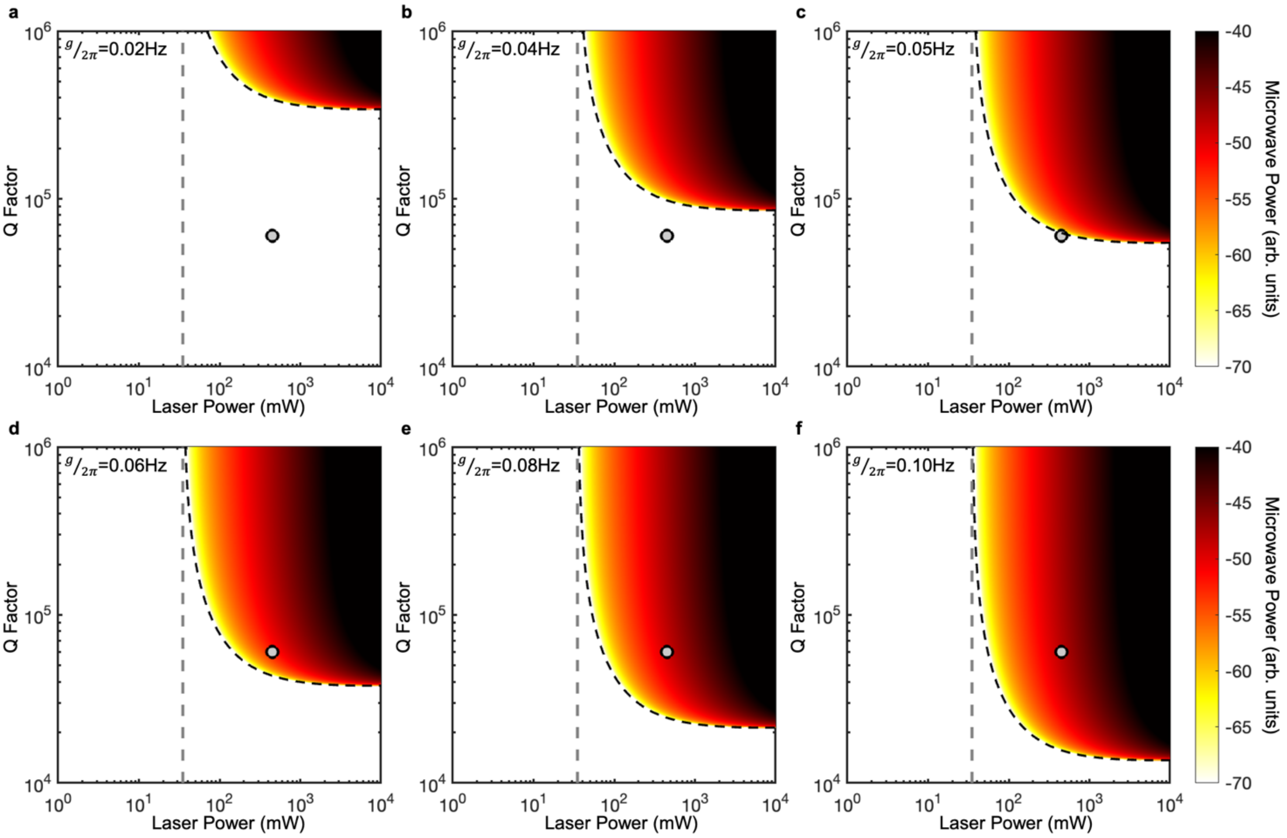

Supplementary Figure 15. Maser simulations for different couplings g. The grey circle illustrates the maser measurement of Figure 1c. Since the output power is very small, we expect the maser threshold to be in this parameter range. We achieve the best agreement with the model for $g/2\pi = 0.05$ Hz which is within the range of other reported values for similar resonators. [4]

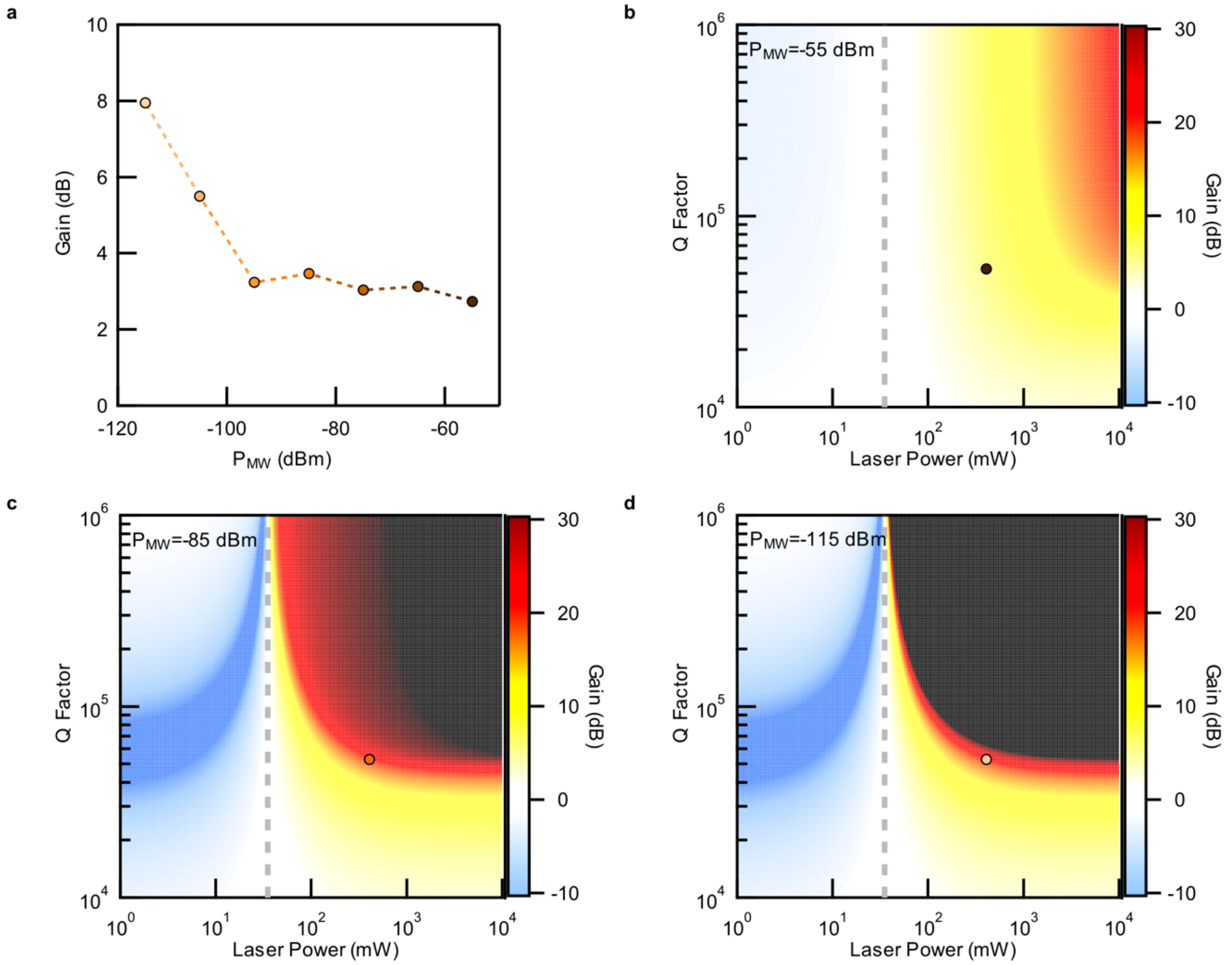

Supplementary Figure 16. Power-dependent SiC-based amplifier. a Measured gain for various input powers. **b-d** Simulations for different microwave input powers (-55 dBm, -85 dBm, -115 dBm). A higher gain is expected for smaller incoming signals, which fits qualitatively to the experimental observation.

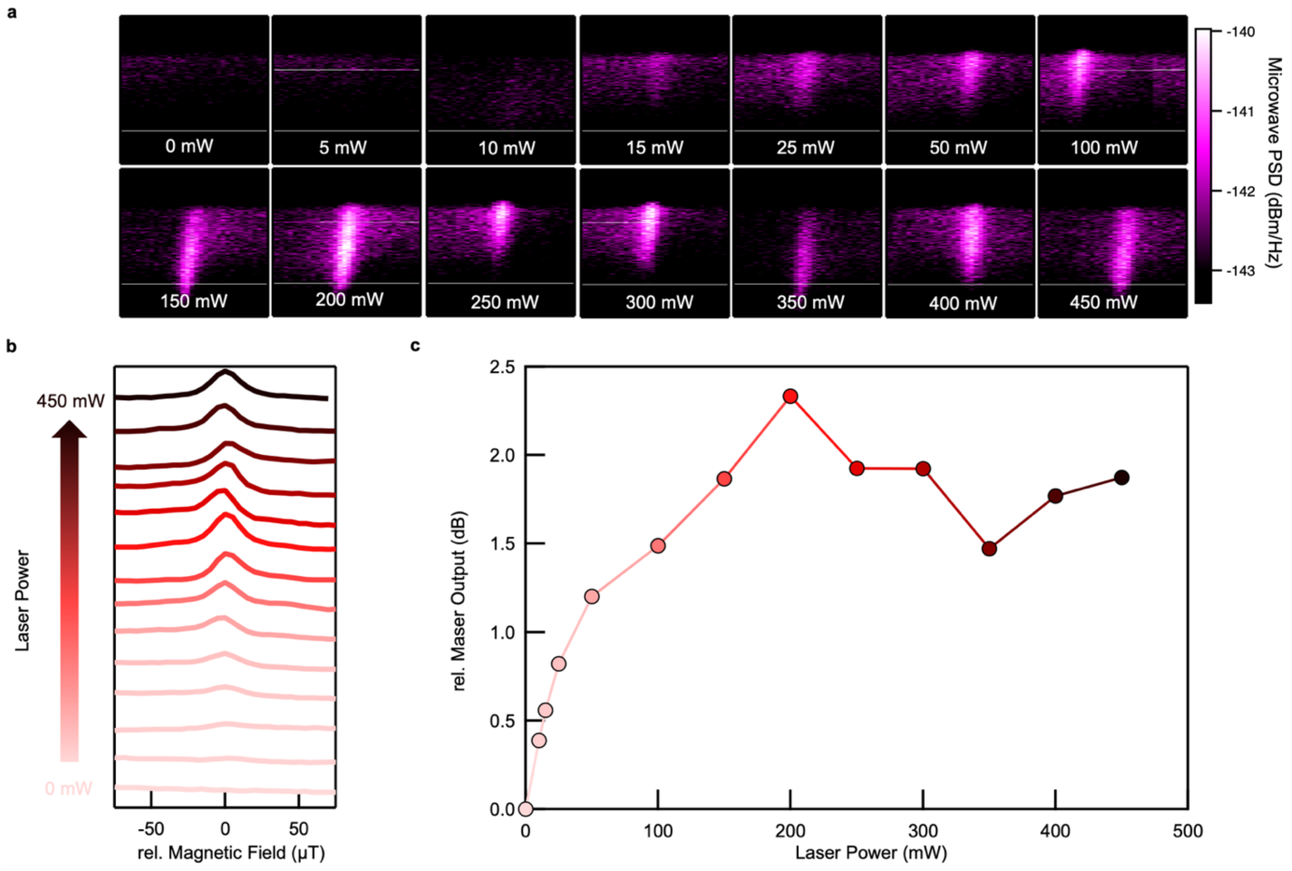

Supplementary Figure 17. Laser power dependence of maser output and its saturation. a Maser measurements without feedback loop at 110 K similar to Figure 1c in the main text. Masing occurs only at higher laser powers and saturates. **b** Qualitative analysis of the maser output by averaging across the different frequencies. **c** Quantitative analysis of the maser output for various laser powers with respect to the background noise. Saturation occurs for multiple hundreds of mW of laser power.

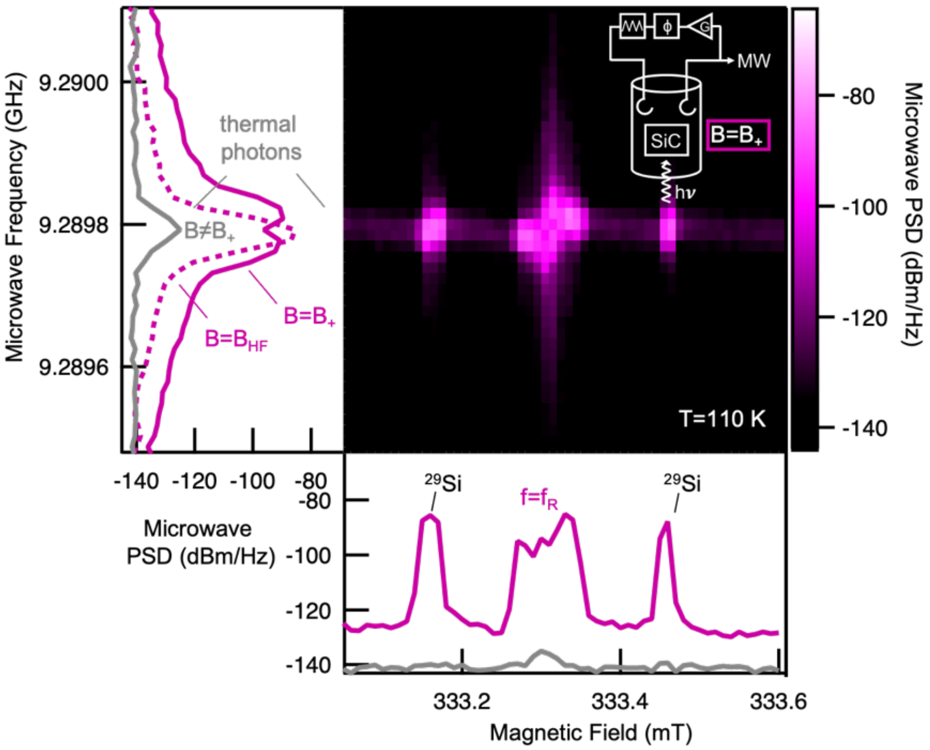

Supplementary Figure 18. Maser saturation for high feedback-loop gain. A strong indicator of saturation is the broadening of the main emission peak, accompanied by the saturation of the hyperfine peak intensities at the same intensity. Additionally, the presence of amplified thermal photons becomes evident, similar to the observation made at 315 K in the main text.

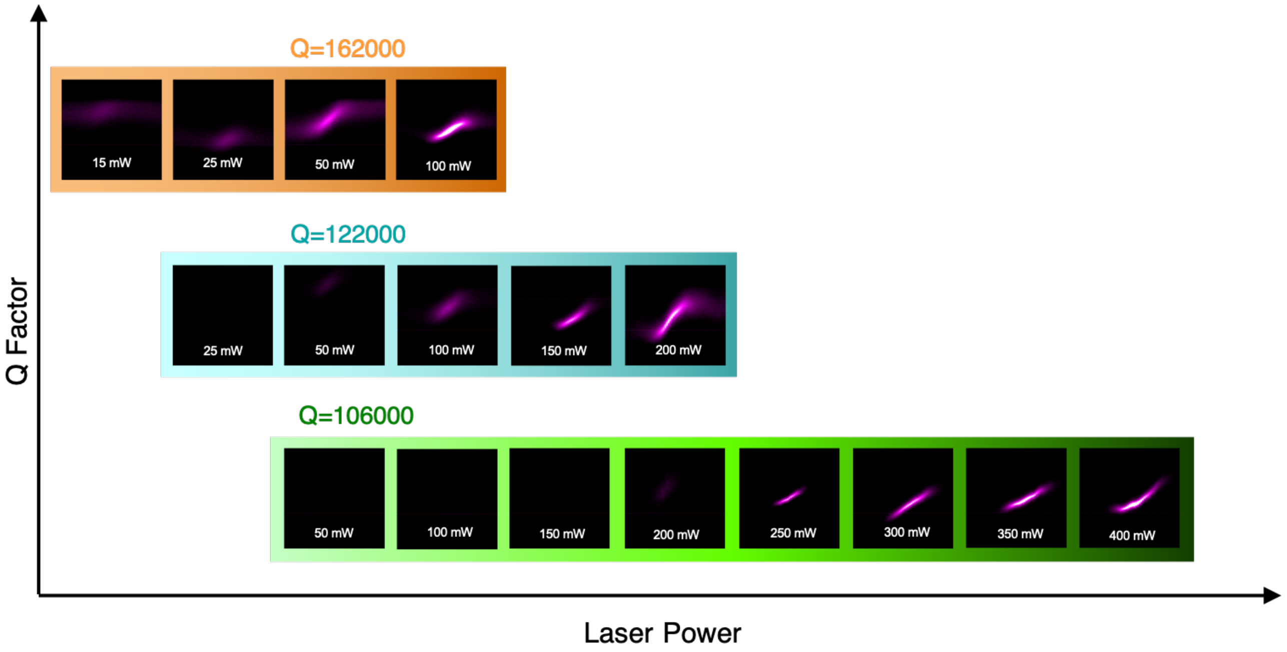

Supplementary Figure 19. Overview of Q-boosted maser measurements for different laser power and Q-factors. This figure summarizes the data sets which were used in the main text in Figure 2c. **a** Maser measurements for Q-factors of 162000 (orange), **b** 122000 (blue) and **c** 106000 (green). The color maps represent the microwave output power (pink) analog to Figure 2b in the main text. As expected from the simulations, masing occurs for higher Q-factors at lower pump powers.

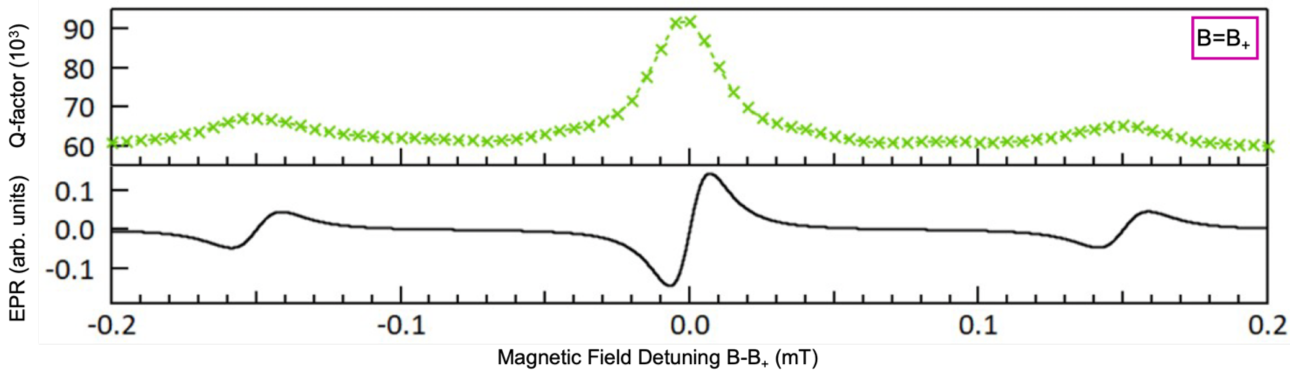

Supplementary Figure 20. Impact of resonant transition on the Q-factor of the resonator. All previously reported Q-factor values are off-resonant (in respect of magnetic field). Once the magnetic field is in resonance with the spin system, losses are reduced due to stimulated emission leading to an enhanced Q-factor of the system.

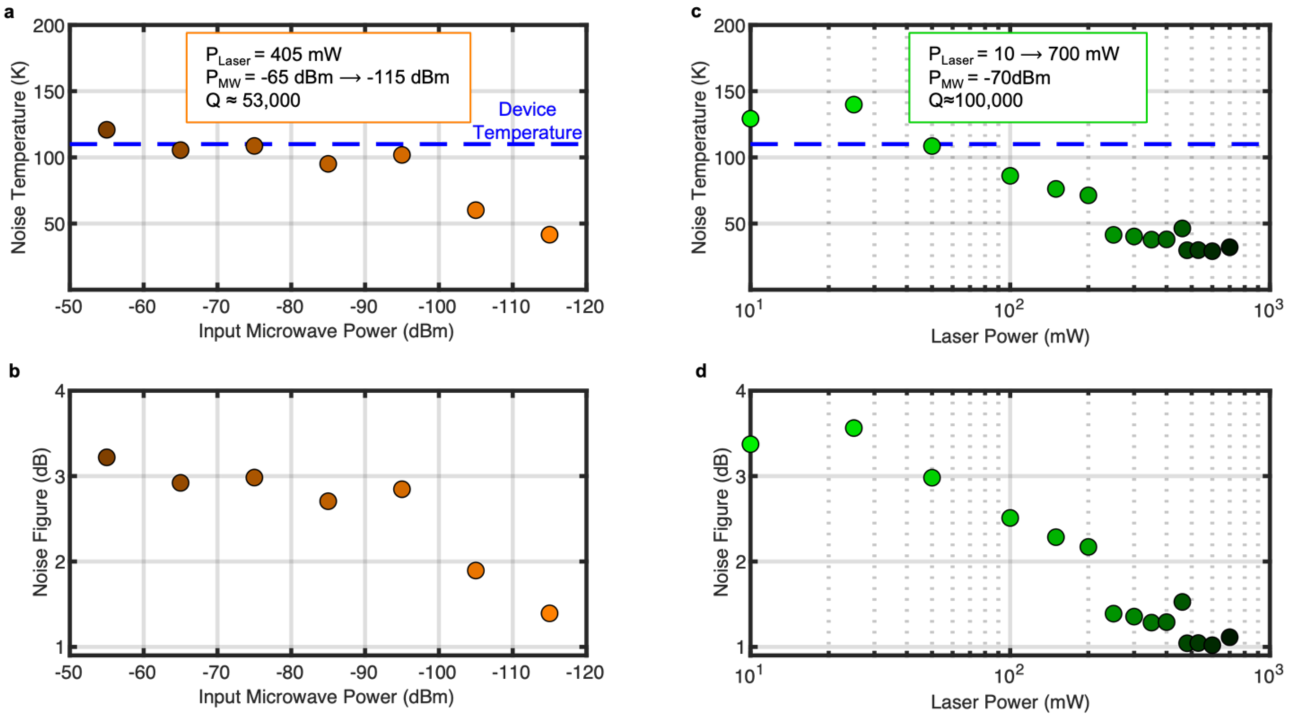

Supplementary Figure 21. Noise performance of maser-based amplifier. The noise temperature can be reduced below the physical device temperature by operating at low input powers and high laser pump powers. The noise figure ranges from 1 dB to 3.4 dB, following a consistent trend. Calculations are based on the model presented in [8].

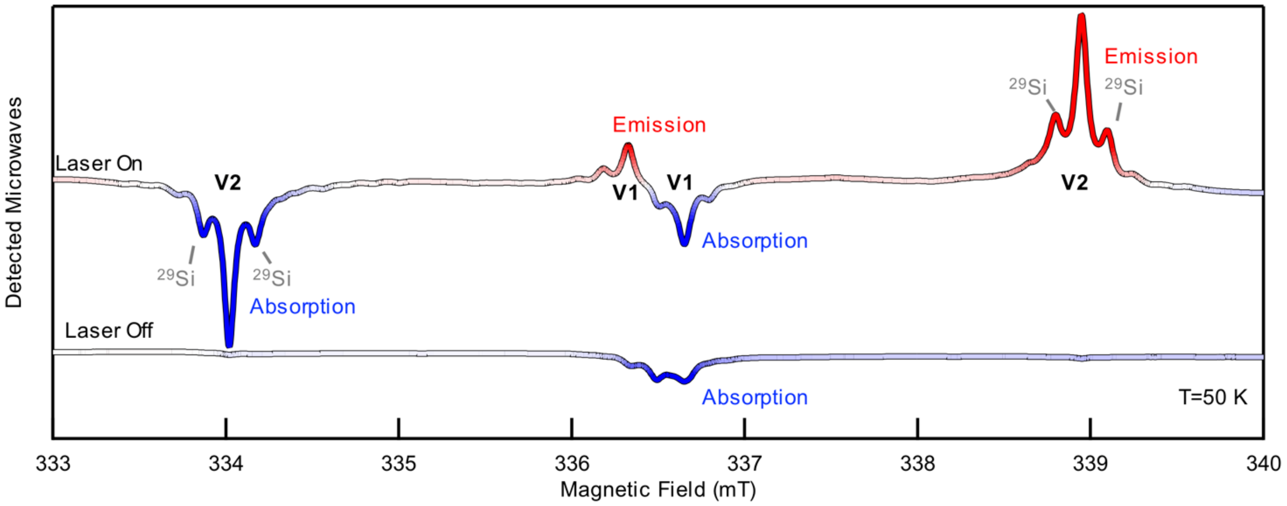

Supplementary Figure 22. Comparison of the silicon vacancies V1 and V2. The detected microwaves (integrated EPR signal) under illumination reveal the absorptive and emissive character of both defects at different magnetic fields. For proof-of-concept measurements, the V2 is favored since the effect of emission and absorption is larger and therefore easier to test for an amplifier/refrigerator. For future application of a magnetic switching of amplifier and microwave cooling devices (e.g. quantum computing) we recommend the V1 defect, since the field difference of the absorptive and emission transition is in the range of $\Delta B \approx 325$ µT due to the smaller ZFS.

Estimation of the Magnetic Field Sensitivity

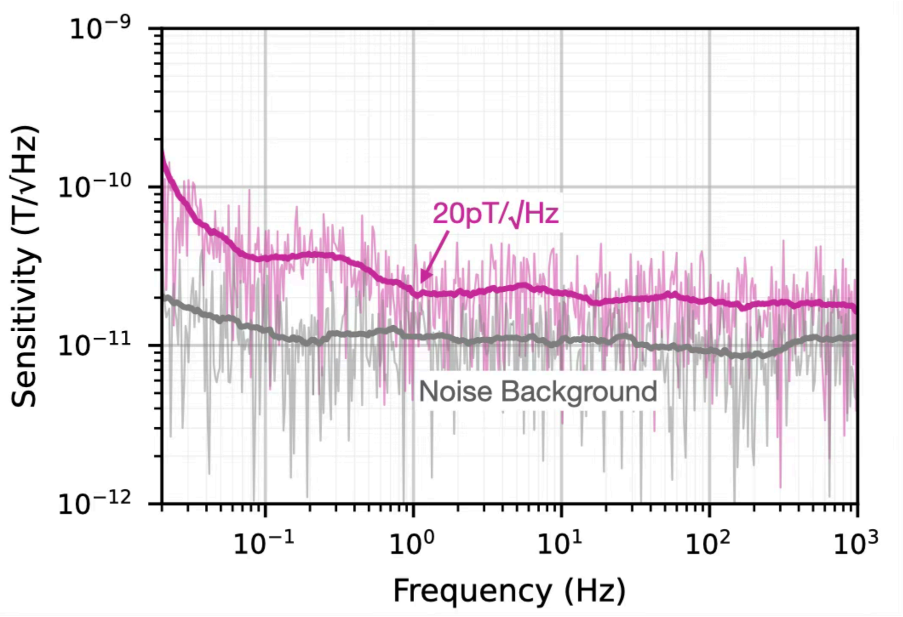

Supplementary Figure 23. Magnetic-field sensitivity of the maser-based magnetometer. The pink curve shows the laser-limited sensitivity estimate derived from the relative intensity noise of the excitation laser (808 nm, K808FANFA-15.00W, BWT), measured in current-sensing mode with a photodetector connected to a Tektronix DPO 3034 oscilloscope. The maser is operated well above threshold, such that small fluctuations around the operating point allow a first-order linear conversion of optical power noise into equivalent signal noise. Using the measured relative laser noise (in 1/√Hz) and the maser linewidth, we infer an estimated magnetic-field sensitivity of 20 pT/√Hz at 1 Hz. Slow temperature-induced drifts of the cavity occur on hour timescales and lie below the measurement bandwidth, while environmental magnetic and microwave-electronics noise are not included, as the figure represents a laser-limited estimate. The grey trace shows the oscilloscope background noise with the laser switched off.

In general, the magnetic field sensitivity of a magnetometer can be written as

$$\frac{\partial B}{\sqrt{\Delta f}} = \frac{\partial S}{\frac{\Delta S}{\Delta B}} \cdot \frac{1}{\sqrt{\Delta f}} = \frac{\Delta B}{\frac{\Delta S}{\partial S}} \cdot \frac{1}{\sqrt{\Delta f}}$$

where S is the detected signal. For an ODMR magnetometer this would be the photoluminescence intensity, while in our case it is the emitted microwave power. For small variations around the resonance condition this expression can be approximated as

$$\frac{\partial B}{\sqrt{\Delta f}} \approx \frac{\Delta B}{\mathrm{SNR}} \cdot \frac{1}{\sqrt{\Delta f}}$$

Here, ΔB denotes the FWHM of the resonance. This follows directly from the linewidth definition: moving away from resonance by the HWHM reduces the signal amplitude to half of its maximum. In addition, the inverse SNR is equal to the relative noise of the detected signal. The key quantity is therefore the relative noise of the detected signal, which can originate from several physical sources.

1) Laser amplitude noise (dominant contribution)

The maser response to laser power is generally nonlinear. However, the sensitivity estimate is derived for small fluctuations around a fixed operating point, where a first-order linear approximation is valid. At our maser operating point, the calibrated laser produces a photocurrent of 47.8 mA in the photodetector (behind an ~OD2 neutral density filter) with a measured noise of 1 μA, corresponding to relative noise of approximately $2 \cdot 10^{-5}$. This small variation justifies the linear approximation used to convert optical power noise into equivalent signal noise. We note that strong nonlinear behaviour occurs close to threshold, where the maser can switch on and off. In contrast, our measurements are performed well beyond threshold.

2) Temperature fluctuations

Temperature variations shift the cavity resonance and can therefore change the detected signal amplitude. The solid copper cavity is buffered with helium gas to stabilize the temperature, and the remaining maser drift occurs only on hour timescales. Consequently, these slow drifts (< mHz) lie below the measurement bandwidth and do not contribute to the quoted sensitivity. While the sensitivity in a 1 Hz bandwidth is therefore only weakly affected, we emphasize in the manuscript that improved frequency stabilization will be required to ensure long-term stability and accuracy.

3) Magnetic-field and microwave-electronics noise

The purpose of the sensitivity estimate reported here is to evaluate the readout-limited sensitivity of the maser. Environmental magnetic noise from the bias magnet and the laboratory environment (including ambient fields such as the Earth's magnetic field) is considered part of the magnetic field the sensor is probed with. A separation of these contributions using calibrated AC magnetic fields has been performed by 9, which is, however, beyond the scope of the present work.

Based on the measured laser noise, we estimate a magnetic-field sensitivity of approximately

$$\frac{\partial B}{\sqrt{\Delta f}} \approx \frac{\Delta B}{\mathrm{SNR}} \cdot \frac{1}{\sqrt{\Delta f}} \approx \frac{1\mu\mathrm{T}}{1/(2 \cdot 10^{-5})} \cdot \frac{1}{\sqrt{1\mathrm{Hz}}} = 20\ \frac{\mathrm{pT}}{\sqrt{Hz}}$$

Supplementary References

1. Fischer, M., Sperlich, A., Kraus, H., Ohshima, T., Astakhov, G. V., & Dyakonov, V. Highly efficient optical pumping of spin defects in silicon carbide for stimulated microwave emission. *Phys. Rev. Appl.* **9**, 054006 (2018).

2. Gottscholl, A., Kianinia, M., Soltamov, V., Orlinskii, S., Mamin, G., Bradac, C., Kasper, C., Krambrock, K., Sperlich, A., Toth, M., Aharonovich, I., & Dyakonov, V. Initialization and read-out of intrinsic spin defects in a van der Waals crystal at room temperature. *Nat. Mater.* **19**, 540-545 (2020).

3. Gottscholl, A., Wagenhöfer, M., Klimmer, M., Scherbel, S., Kasper, C., Baianov, V., Astakhov, G. V., Dyakonov, V. & Sperlich, A. Superradiance of Spin Defects in Silicon Carbide for Maser Applications. *Front. Photonics* **3**, 886354 (2022).

4. Jin, L., Pfender, M., Aslam, N., Neumann, P., Yang, S., Wrachtrup, J., & Liu, R. B. Proposal for a room-temperature diamond maser. *Nat. Commun.* **6**, 1-8 (2015).

5. Simin, D., Kraus, H., Sperlich, A., Ohshima, T., Astakhov, G. V., & Dyakonov, V. Locking of electron spin coherence above 20 ms in natural silicon carbide. *Phys. Rev. B* **95**, 161201 (2017).

6. Lekavicius, I., Myers-Ward, R. L., Pennachio, D. J., Hajzus, J. R., Gaskill, D. K., Purdy, A. P.,Yeats, A.L. Brereton, P. G., Glaser, E. R., Reinecke, T. L. & Carter, S. G. Orders of Magnitude Improvement in Coherence of Silicon-Vacancy Ensembles in Isotopically Purified 4 H-SiC. *PRX Quantum* **3**, 010343 (2022).

7. Breeze, J. D., Salvadori, E., Sathian, J., Alford, N. M., & Kay, C. W. Continuous-wave room-temperature diamond maser. *Nature* **555**, 493-496 (2018).

8. Kraus, H., Soltamov, V. A., Riedel, D., Väth, S., Fuchs, F., Sperlich, A., Baranov, P. G., Dyakonov, D. & Astakhov, G. V. Room-temperature quantum microwave emitters based on spin defects in silicon carbide. *Nat. Phys.* **10**, 157-162 (2014).

9. Eisenach, E. R., Englund, D. R., Barry, J. F. O'Keeffe, M. F., Schloss, J. M., Steinecker, M. H. & Braje, D. A. Cavity-enhanced microwave readout of a solid-state spin sensor. *Nat. Commun.* **12**, 1357 (2021).
