# Supplementary material for: Semiconductor room-temperature maser
Source: Nat Commun. 2026 Jul 25;17:7267. doi: 10.1038/s41467-026-75446-2 (PMC13400640; doi:10.1038/s41467-026-75446-2)
Supplement: Supplementary file 2 — Transparent Peer Review file [file 41467_2026_75446_MOESM2_ESM.pdf]

nature portfolio

Peer Review File

Semiconductor Room-Temperature Maser

Corresponding Author: Dr Andreas Sperlich

This manuscript has been previously reviewed at another journal. This document only contains information relating to versions considered at Nature Communications.

This file contains all reviewer reports in order by version, followed by all author rebuttals in order by version.

Version 0:

Reviewer comments:

Reviewer #1

(Remarks to the Author)
Review of NCOMMS-25-61029-T (revised manuscript)
The authors have made substantial and constructive revisions. Over-stated claims have been tempered, the narrative is better aligned with what is experimentally demonstrated, and the potential impact of the device is presented more realistically. I believe the paper is close to publishable. Below are minor points to address for clarity and completeness.
Remaining points (minor)
1. Magnetometer performance: report calibrated sensitivity and bandwidth
The text compellingly argues for very high contrast (≈ 10^6 %) and a much narrower maser linewidth than ODMR/EDMR, and it introduces a figure-of-merit based on contrast/linewidth. However, there is no calibrated magnetic-field sensitivity (e.g., nT /√Hz) and bandwidth reported for the proposed maser-based magnetometry.

2. Abstract: specify the operating temperature for each performance claim
The abstract states “gain exceeding 10 dB,” but it is not explicit that this value was achieved at 110 K (not at room temperature).

3. Abstract wording: “nine orders of magnitude improvement in spin readout efficiency”
As written, it is unclear what “efficiency” denotes. In the paper, the underlying metric is a proxy—the ratio contrast (C) / linewidth (FWHM)—and the “nine orders” statement refers to this C/FWHM figure-of-merit versus EDMR/ODMR on the same sample type.

4. Context/citations for maser-enabled sensing
Please include a brief connection to prior maser-assisted sensing/magnetometry work (even if non-NV, such as the paper – “Enhanced quantum sensing with room-temperature solid-state masers”) and clarify the novelty relative to NV-maser magnetometry (to my knowledge not yet fully realized). A short sentence plus an appropriate citation will situate the contribution without over-claiming.
5. Editorial/typographical fixes
o Line ~345: “shallow-tones limit” → Schawlow–Townes limitү
o Ensure consistent SI spacing and symbols throughout (e.g., “110 K”, “100 mT”, “μT” rather than “uT”).
o Use “et al.” with a period, and standardize “Schawlow–Townes” spelling everywhere.
o Remove occasional duplicate words and check percent spacing/style per journal guidelines (e.g., “1 %” vs “1%”, but be consistent).

Reviewer #2

(Remarks to the Author)
This manuscript presents results regarding maser operation of a system using silicon vacancy centers in 4H-SiC as a gain medium. Maser operation is shown at 110K with fairly low signal, an increased signal due to Q-boosting the cavity and operation of the maser at room temperature. Amplification and MW mode refrigeration are demonstrated. A magnetometry

mode is proposed though a magnetic sensitivity is not given and the vectorization of this system does not seem to be as straightforward as the authors claim. Importantly for the magnetometry application, the Q-boosting results in a drastic narrowing of the spin resonance lineshape to well below its intrinsic linewidth, which combined with the high contrast of the maser readout leads to a promising performance. There are significant corrections and additions from the previous version of this manuscript which I believe warrant its publication after revisions.

1) Line 89: I believe this should be stimulated, not spontaneous emission.

2) It is slightly unclear to me how you measure 0.4% pump efficiency on line 158. Do obtain data on MW power vs optical pump power (like Fig 2c) and then scale the x-axis of fig 2a by some efficiency that takes you from the theoretical/perfectly efficienct pump power to the real pump power?

3) On line 165: it might be mentioned that isotopic purification would, in addition to increasing the coherence time, increase the effective N of the maser since the V2s with next-nearest neighbor 29Si do not participated in the masing.

4) Lines 292-297 seems to be disjointed. How does the coherence time of the V2 ensemble relate to the noise of the amplifier?

5) In the discussion on the magnetometry operation: a contrast that is 10^6% doesn't make sense to me. Contrast is generally normalized to 1, which seems about what the author's magnetometer is operating at if on vs. off resonance gives a 40dB signal change.
6) The path to vectorize this magnetometer is not obvious. On line 370: this doesn't seem like an appropriate citation since Cochrane et al. used an electrical detection scheme at low magnetic field. A more appropriate citation would be "Vector magnetometry based on S=3/2 electronic spins," Sang-Yun Lee*, et al., Phys. Rev. B 92, 115201 (2015). Even then, vector magnetometry at the "magic angle" would require reading out three separate resonances which isn't too difficult for ODMR based schemes but is very difficult for a MW-cavity, if not impossible due to the reliance of population inversion for the masing.
7) Since the magnetometer seems to me the most promising application of this system, I would encourage the authors to give a magnetic sensitivity of this setup.

Version 1:

Reviewer comments:

Reviewer #1

(Remarks to the Author)
The authors have addressed the remaining concerns, and the paper is now suitable for publication.

Reviewer #2

(Remarks to the Author)
The revised manuscript improves clarity in most cases and addresses many of my previous issues. My main issue with the manuscript is the stated magnetic sensitivity, which was added as a revision. I don't think this manuscript should be published until the issue with the sensitivity is addressed:

1) Minor corrections:
line 80 "relaxes spin dependently"
for clarity state P_th in Eqn 3 is optical pump power threshold

2) It is not clear to me how the quoted magnetic sensitivity is measured. The only measurement given in S23 is the PSD of the attenuated laser (compared to the noise floor of the oscope when the laser is off). The authors then seem to use their calibrated signal response to convert this optical power noise to a magnetic noise. The authors claim that laser amplitude noise is the dominant noise source of their readout which isn't immediately obvious, given that MW noise in their electronics and fluctuations in their bias magnet (mentioned in the summary) may also contribute. Ideally, the authors would perform a similar measurement as "Eisenach, E. R. et al. Cavity-enhanced microwave readout of a solid-state spin sensor. Nature Communications 12, 1357 (2021)" where a calibrated ac magnetic field is applied to the system and then the PSD of the sensor is converted to T/rt(Hz) based off that calibration. If that isn't doable, the authors should explicitly state how they convert that optical noise to magnetic sensitivity, especially since the maser signal response is non-linear as a function of laser power under many conditions (like at 110K in S17).

Version 2:

Reviewer comments:

Reviewer #2

(Remarks to the Author)
The authors have addressed my concerns and the manuscript is good to publish.

Open Access This Peer Review File is licensed under a Creative Commons Attribution 4.0 International License, which permits use, sharing, adaptation, distribution and reproduction in any medium or format, as long as you give appropriate credit to the original author(s) and the source, provide a link to the Creative Commons license, and indicate if changes were made.
In cases where reviewers are anonymous, credit should be given to 'Anonymous Referee' and the source.
The images or other third party material in this Peer Review File are included in the article’s Creative Commons license, unless indicated otherwise in a credit line to the material. If material is not included in the article’s Creative Commons license and your intended use is not permitted by statutory regulation or exceeds the permitted use, you will need to obtain permission directly from the copyright holder.
To view a copy of this license, visit https://creativecommons.org/licenses/by/4.0/

Reply to Reviewers' comments:

Reviewer #1 (Remarks to the Author):

Review of NCOMMS-25-61029-T (revised manuscript)
The authors have made substantial and constructive revisions. Over-stated claims have been tempered, the narrative is better aligned with what is experimentally demonstrated, and the potential impact of the device is presented more realistically. I believe the paper is close to publishable. Below are minor points to address for clarity and completeness.
We thank the Reviewer for the thoughtful evaluation and positive feedback. We are pleased that the revisions have improved the manuscript. Below, we address the remaining minor comments point by point.

Remaining points (minor)
1. Magnetometer performance: report calibrated sensitivity and bandwidth
The text compellingly argues for very high contrast (≈ 10^6 %) and a much narrower maser linewidth than ODMR/EDMR, and it introduces a figure-of-merit based on contrast/linewidth. However, there is no calibrated magnetic-field sensitivity (e.g., nT /√Hz) and bandwidth reported for the proposed maser-based magnetometry.
We thank the Reviewer for this valuable comment. While the contrast-to-linewidth ratio provides a noise-independent benchmark for comparing different spin systems, we fully agree that the calibrated magnetic-field sensitivity is the most relevant figure of merit for practical applications. In our system, the dominant noise source arises from fluctuations in the optical excitation source, as the pump rate primarily determines the intensity noise of the maser signal. Any observed frequency noise can be attributed to magnetic-field instabilities external to the maser itself, rather than intrinsic noise of the device. To quantify the magnetometer performance, we determined the magnetic-field sensitivity under masing conditions (pump power ≈620 mW). The laser output is attenuated and then detected by a Hamamatsu S2281 photodiode. The signal was analyzed using a Tektronix DPO 3034 oscilloscope in current sensing mode, which provides access to detailed noise spectra over a broad frequency range. Based on the measured noise

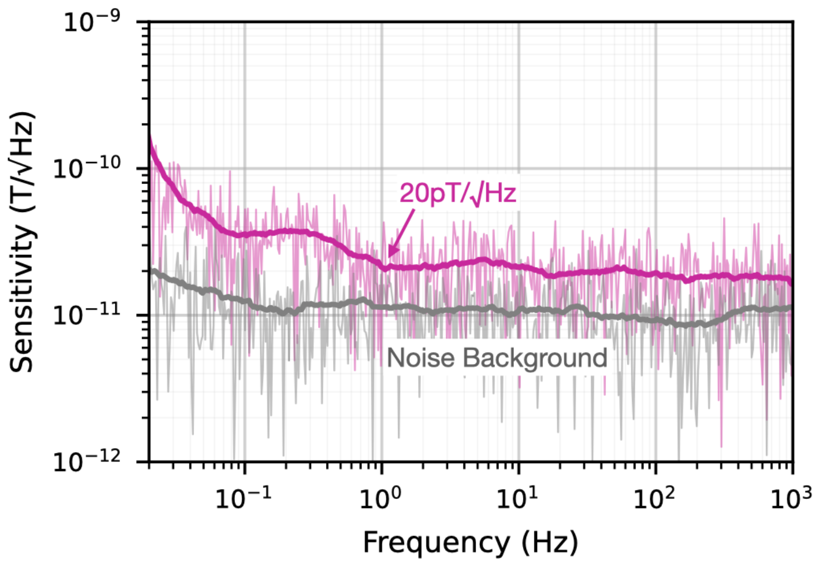

characteristics and the calibrated signal response, a magnetic-field sensitivity of 20 pT/√Hz was determined at a repetition rate of 1 Hz. This exceptional performance is within the expected range of similar systems. We added the value and the comparison to the main text and included the sensitivity measurement in the SI (Fig S23).

2. Abstract: specify the operating temperature for each performance claim
The abstract states “gain exceeding 10 dB,” but it is not explicit that this value was achieved at 110 K (not at room temperature).
We appreciate the comment and have added the operating temperature (110 K) to the abstract to clarify the conditions under which the reported gain (>10 dB) was achieved.

3. Abstract wording: “nine orders of magnitude improvement in spin readout efficiency”
As written, it is unclear what “efficiency” denotes. In the paper, the underlying metric is a proxy—the ratio contrast (C) / linewidth (FWHM)—and the “nine orders” statement refers to this C/FWHM figure-of-merit versus EDMR/ODMR on the same sample type.
We thank the Reviewer for this comment and have clarified in the abstract that the “nine orders of magnitude improvement” refers specifically to the contrast-to-linewidth (C/FWHM) figure of merit, used as a proxy for spin readout efficiency. In addition, following the previous comment, we have also included the measured magnetic-field sensitivity value in the abstract for comparison across different sensing platforms.

4. Context/citations for maser-enabled sensing
Please include a brief connection to prior maser-assisted sensing/magnetometry work (even if non-NV, such as the paper “ –Enhanced quantum sensing with room-temperature solid-state masers”) and clarify the novelty relative to NV-maser magnetometry (to my knowledge not yet fully realized). A short sentence plus an appropriate citation will situate the contribution without over-claiming.
We thank the Reviewer and added a comparison to other maser-assisted sensing work.

5. Editorial/typographical fixes
o Line ~345: “shallow-tones limit” → Schawlow–Townes limitץ
o Ensure consistent SI spacing and symbols throughout (e.g., “110 K”, “100 mT”, “μT” rather than “uT”).
o Use “et al.” with a period, and standardize “Schawlow–Townes” spelling everywhere.
o Remove occasional duplicate words and check percent spacing/style per journal guidelines (e.g., “1 %” vs “1%”, but be consistent).
We thank the Reviewer and have corrected all noted typographical and formatting issues.

Overall, we sincerely thank the Reviewer for the time and effort dedicated to evaluating our manuscript and for the constructive suggestions provided. These comments have significantly improved the clarity, impact, and relevance of our work. We hope that our revisions satisfactorily address all remaining concerns and look forward to any further feedback.

Reviewer #2 (Remarks to the Author):

This manuscript presents results regarding maser operation of a system using silicon vacancy centers in 4H-SiC as a gain medium. Maser operation is shown at 110K with fairly low signal, an increased signal due to Q-boosting the cavity and operation of the maser at room temperature. Amplification and MW mode refrigeration are demonstrated. A magnetometry mode is proposed though a magnetic sensitivity is not given and the vectorization of this system does not seem to be as straightforward as the authors claim. Importantly for the magnetometry application, the Q-boosting results in a drastic narrowing of the spin resonance lineshape to well below its intrinsic linewidth, which combined with the high contrast of the maser readout leads to a promising performance. There are significant corrections and additions from the previous version of this manuscript which I believe warrant its publication after revisions.

We thank the Reviewer for the thorough assessment and constructive suggestions. We are pleased that the revisions strengthen the case for publication. Below, we address each point in detail and indicate the corresponding changes made to the manuscript.

1) Line 89: I believe this should be stimulated, not spontaneous emission.

We thank the reviewer for this comment. We have revised the text to clarify that spontaneous emission provides the initial seed photon for the oscillation, which is subsequently coherently amplified through stimulated emission.

2) It is slightly unclear to me how you measure 0.4% pump efficiency on line 158. Do obtain data on MW power vs optical pump power (like Fig 2c) and then scale the x-axis of fig 2a by some efficiency that takes you from the theoretical/perfectly efficienct pump power to the real pump power?

We thank the Reviewer for this question and have expanded the corresponding section in the manuscript for clarity. The pump efficiency was calculated as the ratio of generated microwave photons to the number of incident optical photons. In our system, approximately 0.4% of the incoming optical photons contribute to microwave photon generation, an inefficient conversion for transducer applications but sufficient to achieve masing. The corresponding power efficiency is even lower, given that the microwave photon energy is over four orders of magnitude smaller than that of an optical photon.

3) On line 165: it might be mentioned that isotopic purification would, in addition to increasing the coherence time, increase the effective N of the maser since the V2s with next-nearest neighbor 29Si do not participated in the masing.

We thank the Reviewer for this insightful observation and have included this additional point in the revised manuscript.

4) Lines 292-297 seems to be disjointed. How does the coherence time of the V2 ensemble relate to the noise of the amplifier?

We thank the Reviewer for this helpful comment and have clarified the paragraph accordingly. The coherence time T_2^* affects the amplifier noise indirectly through its influence on the gain. In this work, we refer to the theoretical framework of Jin et al. (“Proposal for a room-temperature diamond maser”), where the gain is related to the

decay rate of the collective mode ($\approx 2/T_2^*$) as given in equation (57) of the SI. A longer coherence time leads to higher gain, which both reduces the effective noise temperature by L/G (L: losses, G: gain) and brings the amplifier noise temperature closer to the fundamental limit (here: $\hbar\omega_c/k_B \approx$ 0.5 K), as described by equation (27) in Jin et al.

5) In the discussion on the magnetometry operation: a contrast that is 10^6% doesn't make sense to me. Contrast is generally normalized to 1, which seems about what the author's magnetometer is operating at if on vs. off resonance gives a 40dB signal change.
We thank the Reviewer for this valuable comment and have clarified our definition of contrast in the manuscript. In literature, several conventions exist. For example, in ODMR studies of NV centers in diamond, contrast is often normalized to 1, where the off-resonant signal equals 1 and the on-resonant value drops by the contrast (e.g., to 0.9 for a 10% change). Other works, particularly in SiC, define contrast as the relative change with an off-resonance baseline of 0%. We follow this latter definition, as it better reflects the strong on/off switching behavior of the maser signal. If we normalized to 1, our data would correspond to ~40 dB (≈0.01%) contrast, which would misrepresent the relative change compared to typical ODMR in SiC. We have clarified this choice in the revised manuscript to ensure consistency and avoid confusion.

6) The path to vectorize this magnetometer is not obvious. On line 370: this doesn't seem like an appropriate citation since Cochrane et al. used an electrical detection scheme at low magnetic field. A more appropriate citation would be "Vector magnetometry based on S=3/2 electronic spins," Sang-Yun Lee*, et al., Phys. Rev. B 92, 115201 (2015). Even then, vector magnetometry at the "magic angle" would require reading out three separate resonances which isn't too difficult for ODMR based schemes but is very difficult for a MW-cavity, if not impossible due to the reliance of population inversion for the masing.
We thank the Reviewer for this comment and the opportunity to clarify this point. The discussion of vector magnetometry was included only as part of the outlook. Our proposed modulation and demodulation approach, using three modulation frequencies along different directions, could in principle enable vector operation with a single-axis defect, as we have already demonstrated for the same spin system using ODMR. However, since this concept has not yet been experimentally demonstrated for the maser, we have removed this part from the outlook and plan to present it in detail in a follow-up publication.

7) Since the magnetometer seems to me the most promising application of this system, I would encourage the authors to give a magnetic sensitivity of this setup.
We thank the Reviewer for this helpful suggestion and have added corresponding noise measurements to the manuscript. In our system, the dominant noise source originates from fluctuations in the optical excitation, as the pump rate primarily determines the maser's intensity noise. Any observed frequency noise is attributed to magnetic-field instabilities external to the maser rather than intrinsic device noise. To quantify the magnetometer performance, we determined the magnetic-field sensitivity under masing conditions (pump power ≈620 mW) by analyzing the laser noise. The signal was measured using a photodiode and a Tektronix DPO 3034 oscilloscope in current sensing

mode, which provides a noise spectrum over a broad frequency range. From the analysis, a magnetic field sensitivity of 20pT/√Hz was obtained at a repetition rate of 1 Hz. This performance falls within the expected range for comparable systems. The corresponding sensitivity value and its comparison have been added to the main text, and the noise measurement is now included in the Supplementary Information (Fig. S23).

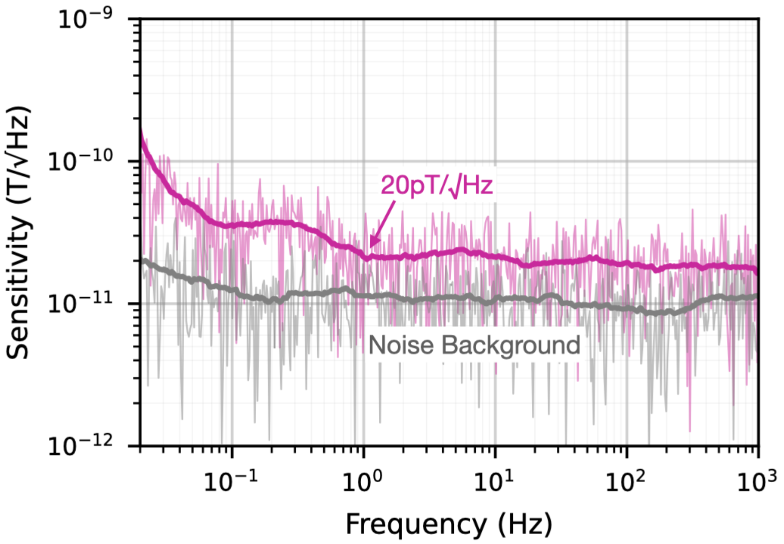

We sincerely thank the Reviewer for the detailed feedback and constructive suggestions, which have greatly strengthened the manuscript. We hope that the additional analyses and measurements provided fully address the raised points and we remain happy to clarify any further questions if needed.

Reply to Reviewers' comments:

Reviewer #1 (Remarks to the Author):

The authors have addressed the remaining concerns, and the paper is now suitable for publication.

We thank the reviewer for the careful evaluation of our manuscript and for the positive assessment. We sincerely appreciate the reviewer's time and commitment during the review process.

Reviewer #2 (Remarks to the Author):

The revised manuscript improves clarity in most cases and addresses many of my previous issues. My main issue with the manuscript is the stated magnetic sensitivity, which was added as a revision. I don't think this manuscript should be published until the issue with the sensitivity is addressed:

We thank the reviewer for this comment. In the revised manuscript we have clarified how the magnetic sensitivity is derived, explicitly stated the assumptions behind the optical-noise-based estimate, and expanded the discussion of the relevant noise sources and limitations.

1) Minor corrections:
line 80 "relaxes spin dependently"
for clarity state P_th in Eqn 3 is optical pump power threshold

We thank the reviewer for this suggestion. We have revised the text accordingly.

2) It is not clear to me how the quoted magnetic sensitivity is measured. The only measurement given in S23 is the PSD of the attenuated laser (compared to the noise floor of the oscope when the laser is off). The authors then seem to use their calibrated signal response to convert this optical power noise to a magnetic noise. The authors claim that laser amplitude noise is the dominant noise source of their readout which isn't immediately obvious, given that MW noise in their electronics and fluctuations in their bias magnet (mentioned in the summary) may also contribute. Ideally, the authors would perform a similar measurement as "Eisenach, E. R. et al. Cavity-enhanced microwave readout of a solid-state spin sensor. Nature Communications 12, 1357 (2021)" where a calibrated ac magnetic field is applied to the system and then the PSD of the sensor is converted to T/rt(Hz) based off that calibration. If that isn't doable, the authors should explicitly state how they convert that optical noise to magnetic sensitivity, especially since the maser signal response is non-linear as a function of laser power under many conditions (like at 110K in S17).

We thank the reviewer for this comment and for the opportunity to clarify how the quoted magnetic sensitivity is obtained as explained below and we added this to the supporting information as well.

In general, the magnetic field sensitivity of a magnetometer can be written as

$$\frac{\partial B}{\sqrt{\Delta f}} = \frac{\partial S}{\frac{\Delta S}{\Delta B}} \cdot \frac{1}{\sqrt{\Delta f}} = \frac{\Delta B}{\frac{\Delta S}{\partial S}} \cdot \frac{1}{\sqrt{\Delta f}}$$

where S is the detected signal. For a conventional ODMR magnetometer this would be the photoluminescence intensity, while in our case it is the emitted microwave power. For small variations around the resonance condition this expression can be approximated as

$$\frac{\partial B}{\sqrt{\Delta f}} \approx \frac{\Delta B}{\mathrm{SNR}} \cdot \frac{1}{\sqrt{\Delta f}}$$

Here, ΔB denotes the FWHM of the resonance. This follows directly from the linewidth definition: moving away from resonance by the HWHM reduces the signal amplitude to half of its maximum. In addition, the inverse SNR is equal to the relative noise of the detected signal. The key quantity is therefore the relative noise of the detected signal, which can originate from several physical sources.

1) Laser amplitude noise (dominant contribution)

The maser response to laser power is generally nonlinear. However, the sensitivity estimate is derived for small fluctuations around a fixed operating point, where a first-order linear approximation is valid. At our maser operating point, the calibrated laser produces a photocurrent of 47.8 mA in the photodetector (behind an ~OD2 neutral density filter) with a measured noise of 1 µA, corresponding to relative noise of approximately 2×10^{-5}. This small variation justifies the linear approximation used to convert optical power noise into equivalent signal noise. We note that strong nonlinear behaviour occurs close to threshold, where the maser can switch on and off. In contrast, our measurements are performed well beyond threshold.

2) Temperature fluctuations

Temperature variations shift the cavity resonance and can therefore change the detected signal amplitude. The cavity is buffered with helium gas to stabilize the temperature, and the remaining maser drift occurs only on hour timescales. Consequently, these slow drifts (< mHz) lie below the measurement bandwidth and do not contribute to the quoted sensitivity. While the sensitivity in a 1 Hz bandwidth is therefore only weakly affected, we emphasize in the manuscript that improved frequency stabilization will be required to ensure long-term stability and accuracy.

3) Magnetic-field and microwave-electronics noise

The purpose of the sensitivity estimate reported here is to evaluate the readout-limited sensitivity of the maser. Environmental magnetic noise from the bias magnet and the laboratory environment (including ambient fields such as the Earth's magnetic field) is considered part of the magnetic field the sensor is probed with. A separation of these contributions using calibrated AC magnetic fields has been performed by [Eisenach et al. 2021], which is, however, beyond the scope of the present work.

Based on the measured laser noise, we estimate a magnetic-field sensitivity of approximately

$$\frac{\partial B}{\sqrt{\Delta f}} \approx \frac{\Delta B}{\mathrm{SNR}} \cdot \frac{1}{\sqrt{\Delta f}} \approx \frac{1\mu\mathrm{T}}{1/10^{-5}} \cdot \frac{1}{\sqrt{1\mathrm{Hz}}} = 20\ \frac{\mathrm{pT}}{\sqrt{Hz}}$$

Verification of this performance would require operation in a magnetically quiet environment using a dedicated shielded facility. Due to the current optical-table-scale implementation of the system, such measurements are not yet feasible. Future work will focus on system miniaturization and measurements in magnetically shielded environments to enable direct calibrated sensitivity measurements. We emphasized in the main text that the sensitivity is estimated based on the laser noise.

The here reported study focuses on several first demonstrations of a semiconductor maser, of which magnetometry represents one application, and we therefore present this result as a first proof-of-concept demonstration.
